# Analysis of Cytoplasmic Effects and Fine-Mapping of a Genic Male Sterile Line in Rice

**DOI:** 10.1371/journal.pone.0061719

**Published:** 2013-04-16

**Authors:** Peng Qin, Yuping Wang, Yuanyuan Li, Bingtian Ma, Shigui Li

**Affiliations:** 1 Rice Research Institute of Sichuan Agricultural University, Chengdu Wenjiang, Sichuan, China; 2 State Key Laboratory of Hybrid Rice, Sichuan Agricultural University, Chengdu Wenjiang, Sichuan, China; Nanjing Forestry University, China

## Abstract

Cytoplasm has substantial genetic effects on progeny and is important for yield improvement in rice breeding. Studies on the cytoplasmic effects of cytoplasmic male sterility (CMS) show that most types of CMS have negative effects on yield-related traits and that these negative effects vary among CMS. Some types of genic male sterility (GMS), including photo-thermo sensitive male sterility (PTMS), have been widely used in rice breeding, but the cytoplasmic effects of GMS remain unknown. Here, we identified a GMS mutant line, *h_2_s*, which exhibited small, white anthers and failed to produce mature pollen. Unlike CMS, the *h_2_s* had significant positive cytoplasmic effects on the seed set rate, weight per panicle, yield, and general combining ability (GCA) for plant height, seed set rate, weight per panicle, and yield. These effects indicated that *h_2_s* cytoplasm may show promise for the improvement of rice yield. Genetic analysis suggested that the phenotype of *h_2_s* was controlled by a single recessive locus. We mapped *h_2_s* to a 152 kb region on chromosome 6, where 22 candidate genes were predicted. None of the 22 genes had previously been reported to be responsible for the phenotypes of *h_2_s*. Sequencing analysis showed a 12 bp deletion in the sixth exon of *Loc_Os06g40550* in *h_2_s* in comparison to wild type, suggesting that *Loc_Os06g40550* is the best candidate gene. These results lay a strong foundation for cloning of the *H_2_S* gene to elucidate the molecular mechanism of male reproduction.

## Introduction

Cytoplasmic male sterility (CMS) is essential to the exploitation of heterosis in three-line hybrid rice breeding programs. CMS cytoplasm has been reported to have substantial genetic effects on various traits of hybrid rice [1], [2], [3]. Studies of the cytoplasmic effects of CMS show that the effects within a type of cytoplasm vary across different agronomic traits in hybrid rice and that the effects on one agronomic trait vary among different types of cytoplasm. For example, the male sterile cytoplasm of *IR19661-283-3-2* has a significant negative effect on both yield and days to flowering but does not affect plant height. The male sterile cytoplasms of *IR46828*, *IR46831*, *IR48483*, *IR54752*, *IR17492-18-10-2-2-3*, *IR54753*, *IR19661-283-1-3-2*, *IR54758*, and *IR54756* have been found to have different effects on grain yield [4]. Most CMS cytoplasms have been reported to have negative effects on yield-related traits, and these effects vary among different sources of cytoplasm [5]. These studies provide practical information for breeding CMS lines and for selecting appropriate CMS line to mate with certain restorers to improve rice yield.

In the three-line system of hybrid rice, male sterility (MS) and its restoration are governed by genetic interaction between male sterile cytoplasm and the nucleus. The interaction maintains the MS, allows the regeneration of the MS line, and restores MS for enjoyment of yield advantage in the F_1_ hybrid. Genetic interaction limits the flexibility of germplasm selection and increases production costs [6]. Genic male sterility (GMS) is solely determined by the nucleus, thus has a wider spectrum of restorers than CMS and facilitates the widespread use of hybrid vigor. GMS can also be used to simplify rice breeding by creating a two-line breeding system. This is because the male sterility caused by recessive GMS can be maintained and restored by introducing a hemizygotic construct into the male sterile plant. This construct comprises a gene whose product is exclusively fatal to pollen and a corresponding restoration gene [7]. Although GMS is useful in rice breeding, the cytoplasmic effects of GMS have not received as much attention as those of CMS. Here, we used a GMS mutant line (*h_2_s*) identified in our breeding program to evaluate cytoplasmic effects on plant height, yield-related traits, and general combining ability (GCA) for these traits.

GMS includes photoperiod-sensitive male sterility (PMS), temperature-sensitive male sterility (TMS), photo-thermo-sensitive male sterility (PTMS), and non-photo-thermo-sensitive genic male sterility. The MS in PMS, TMS, and PTMS can be maintained and reproduced under certain light and temperature conditions. PMS and PTMS have been successfully applied to the production of hybrid seeds, such as Nongken58s, Peiai64s and Y58s [6]. A spontaneous point mutation in *PMS3* (*photoperiod-sensitive male sterility3*) [8] (also called *P/TMS12-1* (*Photoperiod-and-thermosensitive genic male sterility*) [9]) causes PMS in *japonica* background and TMS in *indica* background. Recently, a number of non-photo-thermo-sensitive genic MS genes have been identified in rice, including *WDA1* (*wax-deficient anther1*) [10], *CYP704B2* [11], *TDR* (*tapetum degeneration retardation*) [12], *GAMYB* [13], [14], *DPW* (*defective pollen wall*) [15], and *PTC1* (*persistent tapetal cell1*) [16]. All these genes were found to be related to lipid metabolism and to be critical to anther and pollen exine development. The corresponding mutants show defective anthers and/or pollen development, causing MS in rice. However, the molecular mechanism of anther and pollen development in rice remains unknown. In this study, we identified a male sterile mutant and fine mapped the mutation to a 152 kb region on chromosome 6. Our results lay the groundwork for cloning the *H_2_S* gene and for elucidating the molecular mechanism of anther and pollen development.

## Materials and Methods

### Plant Materials

The *h_2_s* mutant (a spontaneous mutant identified from an *indica* maintainer *Chuannong H_2_S*) and F_2_ mapping populations generated by respectively crossing *h_2_s* as female with Zhenshan97B and *Nipponbare* were planted in a paddy field at Sichuan Agricultural University (Chengdu, Sichuan, China) and at the Hainan rice station (Lingshui, Hainan, China). Five isonuclear alloplasmic lines were generated by crossing heterozygote (*h_2_s*+/−) as male with Zhenshan97A, D702A, G46A, K18A and XieqingzaoA and then backcrossing 8 times using *h_2_s*+/− as the recurrent parent to substitute the nuclei of Zhenshan97A, D702A, G46A, K18A, and XieqingzaoA with the *h_2_s* nucleus. The *h_2_s* mutant was named A1, and the resultant isonuclear alloplasmic lines were A2, A3, A4, A5, and A6, respectively (Table S1). The nuclear background in A1–A6 was checked using 30 ISSR (inter-simple sequence repeat) and 371 SSR markers were used to check the nuclear background of each isonuclear alloplasmic line (A1–A6). A1, A2, A3, A4, A5, and A6 were respectively crossed as female parents with five major restorer lines (Shuhui527 (R1), Minghui63 (R2), Guanghui128 (R3), CDR22 (R4), and Mianhui725 (R5)) to generate 30 F_1_s. For convenience, we defined A1–A6 as parent 1 (P1) and R1–R5 as parent 2 (P2).

### Cytological Characterization of *h2s*

The wild type and the *h_2_s* mutant were cytologically examined for anthers at the meiosis, post-meiosis, young microspore, and vacuolated pollen stages. The preparation for chromosome spreads followed the method described in Chen et al. [17]. Anthers at various stages were fixed overnight in Carnoy’s solution (ethanol:glacial acetic acid = 3∶1) and then rinsed twice with water and twice with 10 mmol/L citrate buffer (pH 4.5), and finally washed with citrate buffer. Treated anthers were placed on a slide with 60% acetic acid and gently pressed with cover glass to release microspore mother cells (MMC). They were then stained with 4′,6-diamidino-2-phenylindole (1 µg/ml DAPI in a buffer containing 50% glycerol and 10 mM citrate, pH 4.5). Paraffin embedding and sectioning were performed using a previous protocol described by Feng et al. [18]. Briefly, the spikelets were fixed overnight in 2.5% glutaraldehyde (pH 6.8) at 4°C and washed twice using 0.1 M phosphate buffer (pH 6.8). They were then dehydrated in an ethanol series and embedded in paraffin (Segma) for polymerization at 45°C for 4 hours. Sections were made using a rotary microtome (Leica, RM2235).

### Experimental Design and Statistical Analysis

Thirty F_1_s and their parents were sown on April 4 and transplanted on May 20, 2006 in the Sichuan Agricultural University paddy (Chengdu, Sichuan, China) to evaluate cytoplasmic effects. A randomized complete block design with three replications was used. For each replication, three rows with 12 plants in each row were planted in plots 2.12 m×0.8 m in size. Rows were 26.6 cm apart and plants within rows were 17.7 cm apart. Ten randomly selected plants from each plot were measured for plant height (cm), effective panicle number, 1,000-grain weight (g), grain number per panicle, seed set rate (%), and weight per panicle (g). Mean values were used for analysis of variance (ANOVA). Most of the plants from each plot were bulk harvested for grain yield, but one plant from each end of each row was omitted to exclude any border effect. This experiment was repeated in the summer of 2008, with the same design. A fixed model with type III sum of squares in SPSS software (Version 21.0. Armonk, NY, U.S.: IBM Corp) was adopted and used to perform an ANOVA. Least significant difference (LSD) was estimated for mean comparison of general combining ability (GCA) effects. The A1–A6 GCA was calculated by the difference between a mean over all combinations for a line A such as A1 to be a parent with all restores (R1–R5) and the overall mean for a trait. The effects of *h_2_s* cytoplasm on plant height and yield-related traits were estimated as follows: [*h_2_s* (A1)/R (R1–R5)] F_1_– [isonuclear alloplasmic line (A2–A6)/R (R1–R5)] F_1_.

### Molecular Mapping

The *h_2_s* gene was mapped using a procedure described previously by Chen et al. [19]. Two F_2_ populations were generated by crossing *h_2_s* as a female with Zhenshan97B and *Nipponbare*, respectively. We used 342 SSR markers to examine polymorphism between *h_2_s* and Zhenshan97B. Preliminary mapping was based on 1532 male sterile F_2_ individuals from the cross between *h_2_s* and Zhenshan97B. For fine mapping, 35 SSRs and 74 indel markers were used to examine polymorphism for the target region between *h_2_s* and *Nipponbare*, and 2400 male sterile F_2_ individuals produced from this cross were analyzed. Among the 74 indel markers, the closest marker to the mutation locus was W11 (F: TTGGTCCCACAAATAAGTCATG; R: TTGGAGCAACTGAAGCAAGGAA). Genetic distance was estimated using Mapmaker 3.0 software [20]. The expression patterns of candidate genes were analyzed using Rice eFP Browser (http://bar.utoronto.ca/efprice/cgi-bin/efpWeb.cgi) and the rice anther expression database [21].

### Expression Analysis for Candidate Gene

Total RNA was isolated using a Qiagen RNA plant mini kit with on-column DNAse digestion (Qiagen). Two micrograms of RNA was used for reverse transcription using M-MLVRT (Promega) with oligo (dT18) primer, and 1 µL RT product diluted with 20 µL ddH_2_O was used as a template for PCR. The RNA used for RT-PCR was from wild type and mutant anthers at the post-meiosis stage, and the primer pair for *Loc_Os06g40550* was W70 (F: CACCACCTACAGTCCCTGCCTCAAA; R: ATGCCGCCCCGCCTATCCTTCCTCA) and *OsActin1* (F: ACGGCGATAACAGCT CCTCTT; R: CCTCTT CCAGCCTTCCTTCAT).

## Results

### Identification and Characterization of *h_2_s*

**Figure 1:**
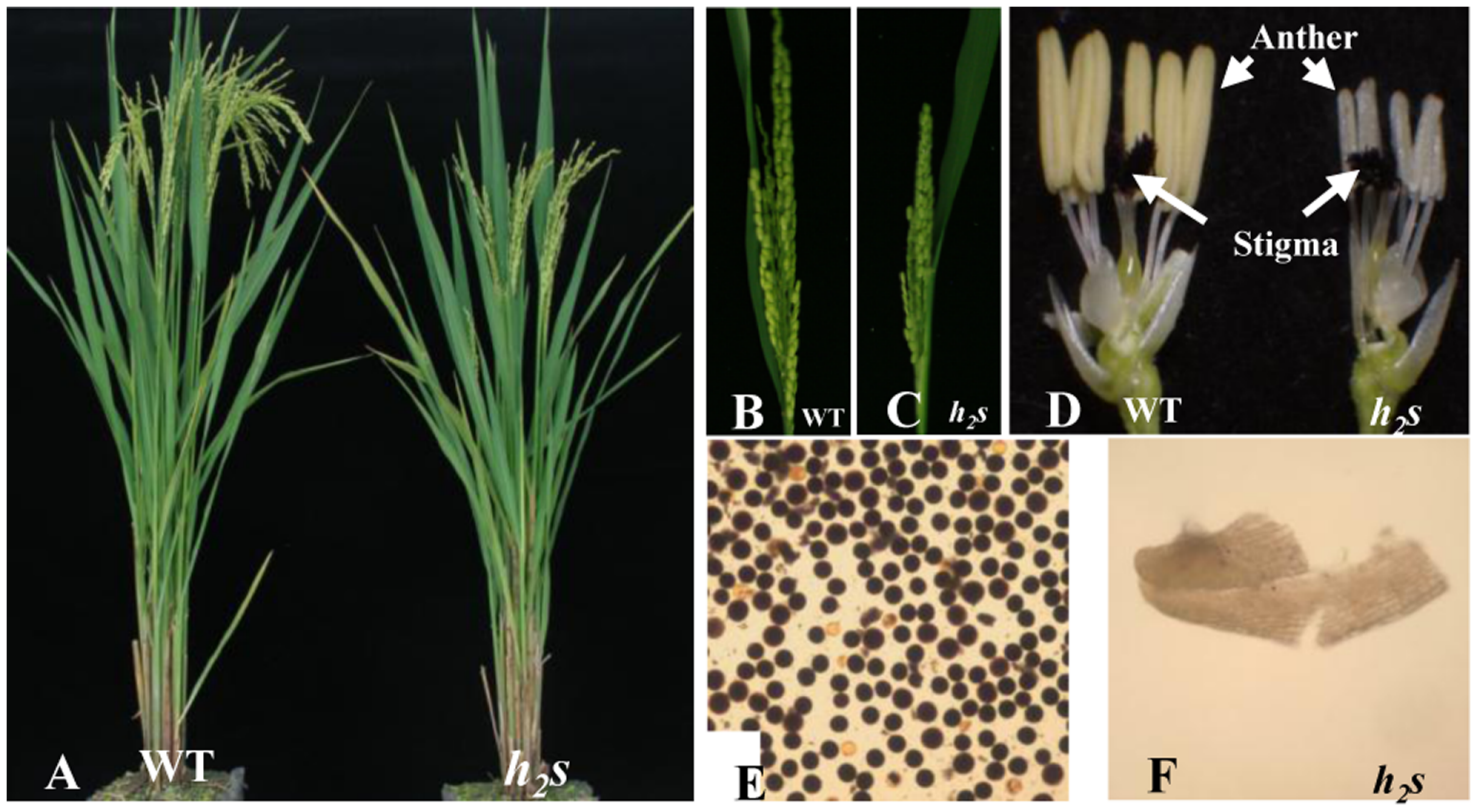
Phenotypic comparison of wild type (WT) and *h_2_s*. Comparison between WT (left) and *h_2_s* (right) for heading plant (A), headed panicle (B, C), flower (D) and pollen of WT (E) and anther of *h_2_s* (F).

**Figure 2:**
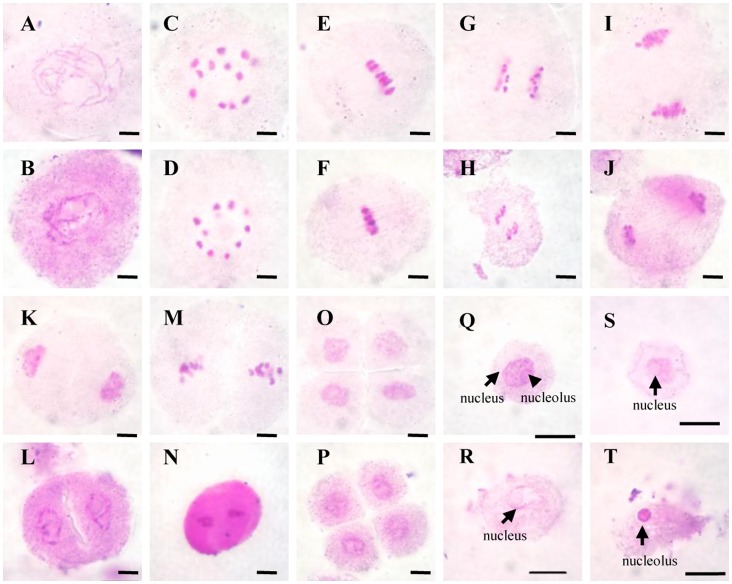
DAPI staining analysis for the meiosis of microspore mother cells and mitosis of young microspores between the wild type (WT) and *h_2_s*. (A–I) and (K–S) for WT, (B–J) and (L–T) for *h_2_s*; (A) and (B) Pachytene, (C) and (D) diakinesis, (E) and (F) metaphase I, (G) and (H) anaphase I, (I) and (J) telophase I, (K) and (L) prophase II, (M) and (N) metaphase II, (O) and (P) tetrad, (Q) and (R) young microspore stage, and (S) and (T) vacuolated pollen stage. No difference in the process of meiosis was observed between WT and *h2s*. Bars = 10 µm.

The *h_2_s* mutant showed shorter spikelets with smaller anthers than the wild type (Figure 1B, C, D) but exhibited normal pistil and vegetative development with only slightly shorter plant height (Figure 1A). The *h_2_s* mutant failed to produce mature pollen (Figure 1E and F). To characterize defects during anther development in the *h_2_s* mutant, we performed 4′, 6-diamidino-2-phenylindole staining. No abnormal activity was observed in *h2s* during meiosis (Figures 2A–P). However, the cytoplasm of the young *h_2_s* microspores collapsed after they were released from tetrads (Figure 2Q and R). At the vacuolated pollen stage, the microspores were completely collapsed and only nuclei were left with some residual cytoplasm in the locule (Figure 2S and T). These observations suggest that the developmental defect in the *h_2_s* mutant occurred after the release of young microspores from tetrads.

**Figure 3:**
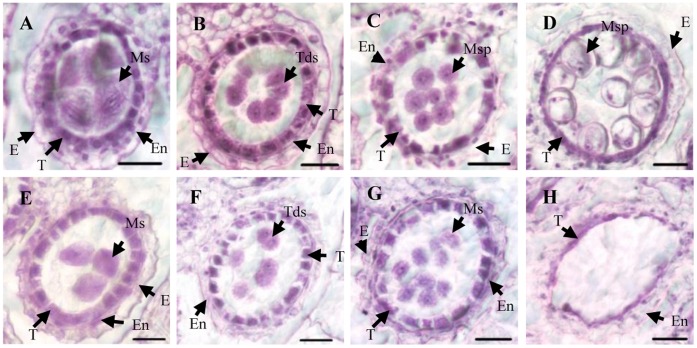
Section analysis between the wild type (WT) and *h_2_s.* (A–D) for WT, (E–G) for *h2s*. (A) and (E) Meiosis, (B) and (F) post-meiotic stage, (C) and (G) young microspore stage. The young microspores in *h2s* exhibited defects at the post-meiotic stage (D) and vacuolated pollen stage (H). However, no microspore was observed at the vacuolated pollen stage. E: epidermis; En: endothecium; T: tapetum; Ms: microsporocyte; Msp: microspore; Tds: tetrad; bars = 15 µm.

To obtain more information regarding the morphological defects of *h_2_s* anthers, we examined thin sections of wild-type and *h_2_s* anthers. At the meiosis stage, as with 4′, 6-diamidino-2-phenylindole staining, no obvious difference between the wild type and the *h_2_s* mutant was observed (Figure 3A and E). At the post-meiotic stage, MMC went through meiosis and formed tetrads in the wild type (Figure 3B). In this way, young microspores in tetrads were released in the anther locule at the young microspore stage (Figure 3C). At the vacuolated pollen stage, the microspore nucleus was pressed near the cytoplasmic membrane by the vacuole and underwent mitosis twice to form mature pollen grains. The tapetum became condensed and degraded (Figure 3D). In contrast, at the post-meiotic stage, the *h_2_s* mutant showed young microspores but there was no clear boundary around them (Figure 3F). At the young microspore stage, as with 4′, 6-diamidino-2-phenylindole staining, the young *h_2_s* microspores appeared degraded. The microspores in *h_2_s* seemed to lack exine, so the microspore degradation might be due to the absence of exine. At the vacuolated pollen stage, the microspores in *h_2_s* were completely degraded and formed empty anther locules.

### Genetic Analysis of the *h_2_s* Mutant

All F_1_ plants from the cross between *h_2_s* and *9311* produced normal mature pollen and seed sets. Among 120 F_2_ plants, 87 had normal pollen and 33 had no pollen, (χ^2^[3∶1] = 0.259; *P*>0.5), indicating a single recessive mutation in the spontaneous *h_2_s* mutant. *h_2_s* also showed stable MS in both Sichuan (E103°82′, N30°97′) and Hainan (E110°05′, N18°28′), suggesting that *h_2_s* was not a PTMS.

### Cytoplasmic Effects of *h_2_s* on Plant Height and Yield-related Traits

No differences in genetic background among the six isonuclear alloplasmic lines (A1–A6) were detected (Figure S1), indicating that the nuclei of Zhenshan97A, D702A, G46A, K18A, and XieqingzaoA were successfully substituted by the *h_2_s* nucleus. This allowed reliable comparison of the cytoplasmic effects among A1–A6.

The combined ANOVA for the data collected in two years (Figures S2, S3, S4, S5, S6, S7, and S8) showed that the cytoplasm (P1) had significant effects on all investigated traits except effective panicle number (Table 1). The year effect was significant on all traits except the 1000-grain number and grain number per panicle. The interaction between year and cytoplasm showed a significant effect on effective panicle number, grain number per panicle, and yield. The cytoplasmic effects of *h_2_s* on plant height and yield-related traits were estimated by comparing two hybrids with the same male parent. Out of 25 paired comparisons, all but two showed that *h_2_s* cytoplasm had a positive effect on yield. These positive effects were significant in 12 (48%) and 10 (40%) paired comparisons in 2006 and 2008, respectively (Table 2). Similarly, all but two paired comparisons showed that *h_2_s* cytoplasm had positive effects on the seed set rate. Thirteen (52%) and fourteen (56%) of such comparisons were found to be significant in 2006 and 2008, respectively. Six (24%) and seven (28%) paired comparisons showed significant positive cytoplasmic effect on weight per panicle in 2006 and 2008, respectively. For plant height, effective panicle number, and 1000-grain weight, there were fewer comparisons with significant positive cytoplasmic effects, even though the high proportion of paired comparisons showed positive cytoplasmic effects (Table S2). In contrast, 4 and 9 paired comparisons showed significant negative cytoplasmic effects on grain number per panicle in 2006 and 2008, respectively (Table S2). In conclusion, the cytoplasm of *h_2_s* showed a significant and positive effect on yield, seed set rate, weight per panicle, and a significant and negative effect on the grain number per panicle, but plant height, 1000-grain weight, and grain number per panicle were less affected.

**Table 1:** Variance analysis of yield-related traits and plant height (F value) for experiments conducted in 2006 and 2008 in Chengdu, China.

Source of variation	Df	Plantheight	Effectivepanicle No.	1,000- grainweight	Grain No./panicle	Seed setrate	Weight/panicle	Yield
Replication	2	3.02	1.95	1.03	2.53	0.30	1.06	0.58
P1(A1–A6)	5	1.82*	1.52	2.15*	3.99**	5.52**	4.97**	11.16**
P2(R1–R5)	4	19.74**	16.19**	71.00**	8.25*	5.13**	65.70**	32.48**
Year	1	34.84**	8.15*	3.20	3.13	9.15*	11.48**	10.73*
P1×P2	20	11.92*	1.60	1.21	3.40**	4.67**	5.44**	3.3**
P1×Year	5	2.44	5.60**	1.554	4.27**	0.48	0.90	4.51*
P2×Year	4	2.08**	4.56**	6.07**	1.32	25.54**	12.03**	11.32**
P1×P2×Year	20	7.47**	0.67	0.18	8.23**	5.38*	1.12	3.14
Error (mean square)	118	806	54	67	4383	1124	2.60	0.34

* and **Significant at 0.05 and 0.01 probability level, respectively.

P1: Isonuclear alloplasmic lines (A1–A6), represents the cytoplasmic effect.

P2: Restorer lines (R1–R5).

**Table 2:** *h_2_s* cytoplasmic effects on seed set rate, weight/panicle and yield in comparison with Zhenshan97A (A2), D702A (A3), G46A (A4), K18A (A5) and XieqingzaoA (A6).

Paired comparison	Seed set rate (%)	Weight/panicle (g)	Yield (kg/plot)
A1/R1–A2/R1	3.94**/4.24**	0.34*/0.30*	0.10*/0.15*
A1/R1–A3/R1	3.64*/6.19**	0.06/−0.05	0.12*/0.13*
A1/R1–A4/R1	5.91**/8.15**	0.21/0.10	0.15**/0.21**
A1/R1–A5/R1	4.05**/6.82**	0.46**/0.42**	0.16**/0.23**
A1/R1–A6/R1	3.61*/6.35**	0.24/0.07	0.12*/0.20**
A1/R2–A2/R2	3.94**/5.01**	0.22/0.20	0.07/0.09*
A1/R2–A3/R2	3.25*/3.54*	0.24/0.14	0.07/0.05
A1/R2–A4/R2	1.07/−1.21	0.25/0.34*	0.05/0.08
A1/R2–A5/R2	0.76/3.36	0.02/−0.06	0.06/0.04
A1/R2–A6/R2	2.52/6.29	0.24/0.35*	−0.04/−0.04
A1/R3–A2/R3	−0.8/1.02	0.08/0.02	0.08/0.07
A1/R3–A3/R3	0.58/1.58	−0.10/−0.06	0.10*/0.08*
A1/R3–A4/R3	1.29/4.77*	−0.07/0.07	0.12**/0.15**
A1/R3–A5/R3	1.76/0.25	0.09/0.11	0.10*/0.07
A1/R3–A6/R3	−0.67/−0.40	0/0.03	0.06/0.06
A1/R4–A2/R4	5.54**/12.42**	0.15/−0.03	0.10*/0.05
A1/R4–A3/R4	8.00**/12.51**	0.41**/0.44**	0.12**/0.09*
A1/R4–A4/R4	8.07**/12.77**	0.09/0.09	0.07/−0.02
A1/R4–A5/R4	7.01**/13.82**	0.39**/0.41**	0.10*/0.09*
A1/R4–A6/R4	10.01**/13.41**	0.43**/0.44**	0.09*/0
A1/R5–A2/R5	0.88/−2.09	−0.02/0.09	0.05/0.02
A1/R5–A3/R5	2.00/0.57	0.33**/0.20	−0.01/0
A1/R5–A4/R5	3.06*/3.22*	0.08/0	0.01/0
A1/R5–A5/R5	0.69/−1.90	−0.09/−0.24	0.07/0.03
A1/R5–A6/R5	2.20/−0.88	0.15/0.09	0.06/0.05
Percentage of comparisons withsignificant positive effect (%)	52/56	24/28	48/40

The numbers separated by slashes represent data from different years, 2006 (left) and 2008 (right). *and **Significant at 0.05 and 0.01 probability level, respectively.

### Cytoplasmic Effects of *h_2_s* on General Combining Ability (GCA)

Studies on GCA have provided information on the potential of specific rice lines as parents in breeding programs. Compared with isonuclear alloplasmic parents A2, A3, A4, A5, and A6, *h_2_s* showed significant positive cytoplasmic effects on GCA with respect to yield-related traits (seed set rate, weight per panicle, and yield) (Table 3) and plant height (Table S3) in both years. However, no cytoplasmic effect on GCA with respect to effective panicle number or grain number per panicle was observed for *h_2_s* in either year (Table S3). The significant and positive cytoplasmic effect on GCA, especially with respect to yield, suggests that the *h_2_s* mutant is a good parent for rice breeding.

**Table 3:** GCA effects on seed set rate, weight per panicle and yield among cytoplasms of *h_2_s* (A1), Zhenshan97A (A2), D702A (A3), G46A (A4), K18A (A5) and XieqingzaoA (A6).

Parents	Seed set rate (%)	Weight/Panicle (g)	Yield (kg/plot)
A1	2.74a/3.79a	0.14a/0.11a	0.07a/0.06a
A2	0.04b/0.07b	−0.01b/0b	−0.01bc/−0.01b
A3	−0.75b/−1.08b	−0.05b/−0.02b	−0.01bc/−0.01b
A4	−1.13b/−1.75b	0.03b/0b	−0.01bc/−0.02b
A5	−0.11b/0.12b	−0.03b/−0.01b	−0.03c/−0.02b
A6	−0.79b/−1.16b	−0.07b/−0.08b	0.01b/0.01b

The numbers separated by slashes represent the data from different years, 2006 (left) and 2008 (right). Values followed by the same letter in a column within a year are not significantly different at α = 0.05.

### Molecular Mapping of the *h_2_s* Mutation

Although cytoplasmic analysis of agronomic traits and GCA indicated that *h_2_s* had considerable potential for the improvement of rice yield, the male sterility of *h_2_s* severely restricts its utility in the production of hybrid seeds. This is because *h_2_s* is a non-photoperiod/thermo-sensitive genic male sterile mutant. However, one previously described approach provides a possibility to maintain the MS of recessive GMS, so that *h_2_s* could be used to produce hybrid seed in breeding practices [7]. This approach requires cloning the gene responsible for MS. For this reason, we tried to clone the *H_2_S* gene using a mapping-based approach.

**Figure 4:**
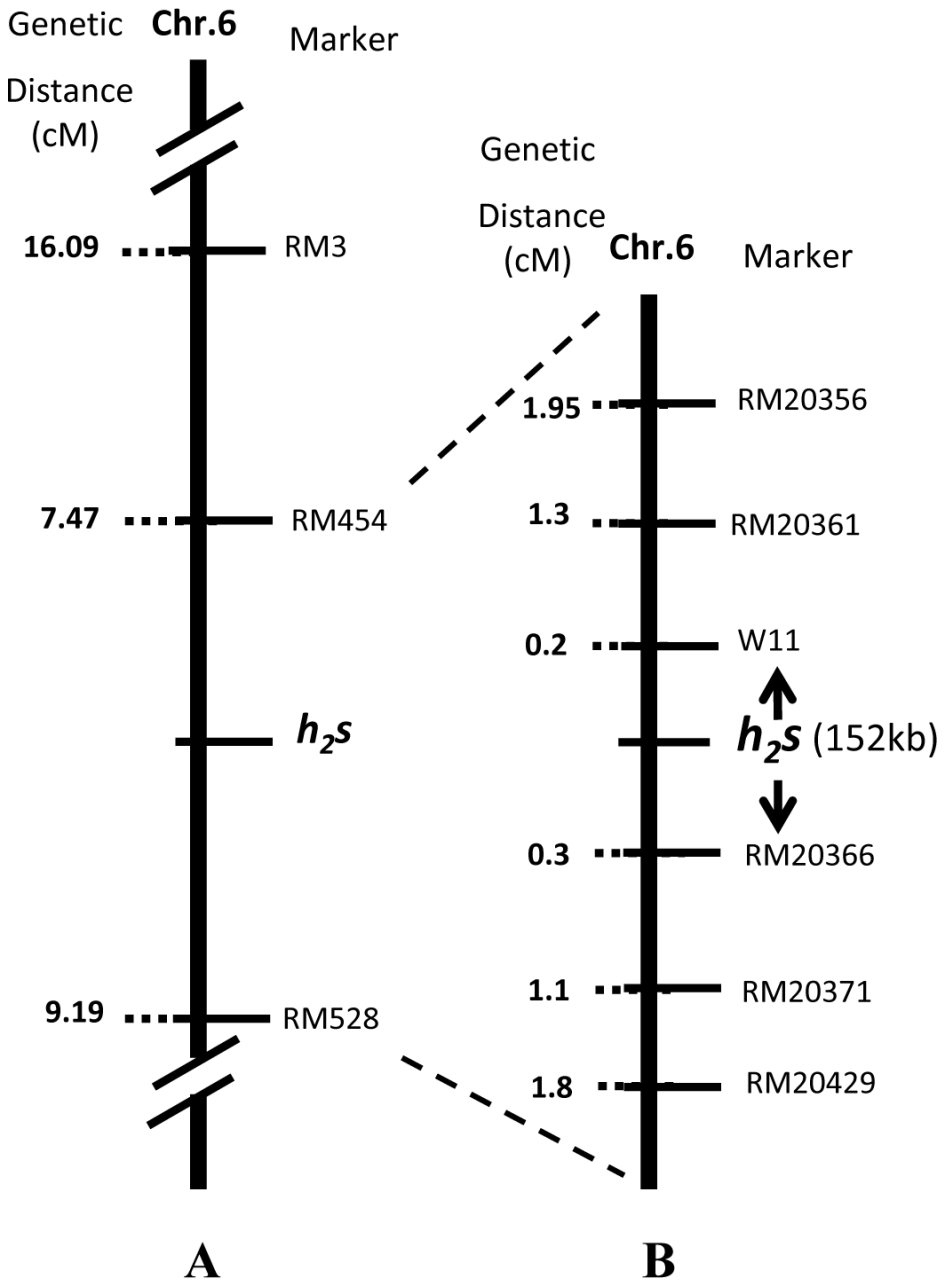
Linkage mapping of the *h_2_s* gene. (A) Preliminary mapping results using a population of ***h_2_s***/Zhensan97B and (B) fine mapping results using a population of ***h_2_s***/Nipponbare. Genetic distance between a marker and the *h_2_s* gene is given on the left in centimorgans (cM).D.

A F_2_ population was generated from a cross between *h_2_s* and Zhensan97B to map the *h_2_s* gene. A bulk segregant analysis was conducted using 52 polymorphic SSR markers between *h_2_s* and Zhensan97B with two DNA bulks, each with ten sterile F_2_ plants and ten fertile F_2_ plants. Of the 52 SSRs, only RM3 on chromosome 6 was polymorphic between the two DNA bulks, indicating that the putative *h_2_s* gene was located on chromosome 6. We then examined 23 SSRs flanking RM3 for polymorphisms between *h_2_s* and Zhensan97B, and the polymorphic SSRs were used to analyze 612 male sterile F_2_ individuals. Both RM454 (located at 27832 kb) and RM528 (located at 26544 kb) were found to be linked with the *h_2_s* locus, with 7.47 and 9.19 cM genetic distance, respectively (Figure 4A). Because no other SSRs were found to be polymorphic between *h_2_s* and Zhensan97B for the region between RM454 and RM528, 35 SSRs and 74 indel markers were examined for polymorphism between *h_2_s* and *Nipponbare*. The identified polymorphic markers were used to analyze 2400 male sterile F_2_ individuals in the cross between *h_2_s* and *Nipponbare*. The *h_2_s* locus was mapped to a 152 kb region between indel W11 (located at 24044 kb) and RM20366 (located at 24196 kb) with 0.2 and 0.3 cM genetic distance, respectively (Figure 4B). A total of 22 genes were predicted in this mapped region [22] (Table 4), and none of them have been previously reported for the *h_2_s* phenotype. This result suggests that *H_2_S* is a novel gene controlling post-meiotic anther and pollen development.

**Table 4:** Candidate genes in the 154 kb region on chromosome 6 where *h_2_s* locus was fine mapped.

Gene ID	Putative function
LOC_Os06g40480	retrotransposon protein
LOC_Os06g40490*	glycosyl hydrolases family 17
LOC_Os06g40500*	Unknown
LOC_Os06g40510	CRAL/TRIO domain containing protein
LOC_Os06g40520	TNP1 domain containing protein
LOC_Os06g40530	transposon protein CACTA, En/Spm sub-class
LOC_Os06g40540*	retrotransposon protein
LOC_Os06g40550*	ABC-2 type transporter domain containing protein
LOC_Os06g40560	26S protease regulatory subunit S10B
LOC_Os06g40570	GRAM and C2 domains containing protein
LOC_Os06g40580*	Unknown
LOC_Os06g40590	Unknown
LOC_Os06g40600	elongation factor
LOC_Os06g40609*	Unknown
LOC_Os06g40620*	SNF7 domain containing protein
LOC_Os06g40630*	SFT2 domain containing protein
LOC_Os06g40640	fructose-bisphospate aldolase isozyme
LOC_Os06g40650*	copine-1
LOC_Os06g40660*	retrotransposon protein
LOC_Os06g40670	unknown
LOC_Os06g40680	unknown
LOC_Os06g40700	OsSub50 - Putative Subtilisin homologue

* expressed in inflorescence.

### Analysis of Candidate Genes

Analysis of expression patterns for the 22 candidate genes showed that ten are expressed in the inflorescence [23] (Table 4). Among the ten candidates, *Loc_Os06g40490* encodes a glycosyl hydrolase that is likely to be involved in carbohydrate metabolic processes [24]. *Loc_Os06g40550* encodes a protein with an ATP binding cassette (ABC) transporter domain. Its homolog in *Arabidopsis*, *ABCG26/WBC27*, has been reported to be involved in pollen exine formation, and the *abcg26/wbc27* mutant exhibits impaired pollen wall formation that causes MS in *Arabidopsis* [25], [26], [27]. The homolog of *SNF7* (*sucrose non-fermenting 7*) protein encoded by *Loc_Os06g40620* in *Arabidopsis* is a subunit of the ESCRT III complex. It may be involved in vesicle-mediated transport [28]. *Loc_Os06g40630* encodes a protein containing a *SFT2* domain, its homolog in yeast is involved in trafficking to the Golgi complex [29]. *Loc_Os06g40650* encodes copine-1, whose homolog in *Arabidopsis* (*ring domain ligase 2*) negatively regulates drought stress response [30]. Two genes (*LOC_Os06g40540* and *LOC_Os06g40660*) are predicted to be retro-transposons, and the genetic functions of three other genes (*LOC_Os06g40500*, *LOC_Os06g40580*, and *LOC_Os06g40609*) are unknown at present. Among the ten candidate genes, *Loc_Os06g40550* is the only one to be expressed in anthers around the tetrad stage [21]. Considering that a mutant of the *Loc_Os06g40550* homolog in *Arabidopsis*, *abcg26/wbc27*, also exhibits MS, and the spatial and temporal expression of *Loc_Os06g40550* was consistent with *h_2_s* phenotype, we chose *Loc_Os06g40550* as a top candidate gene for further study.

**Figure 5:**
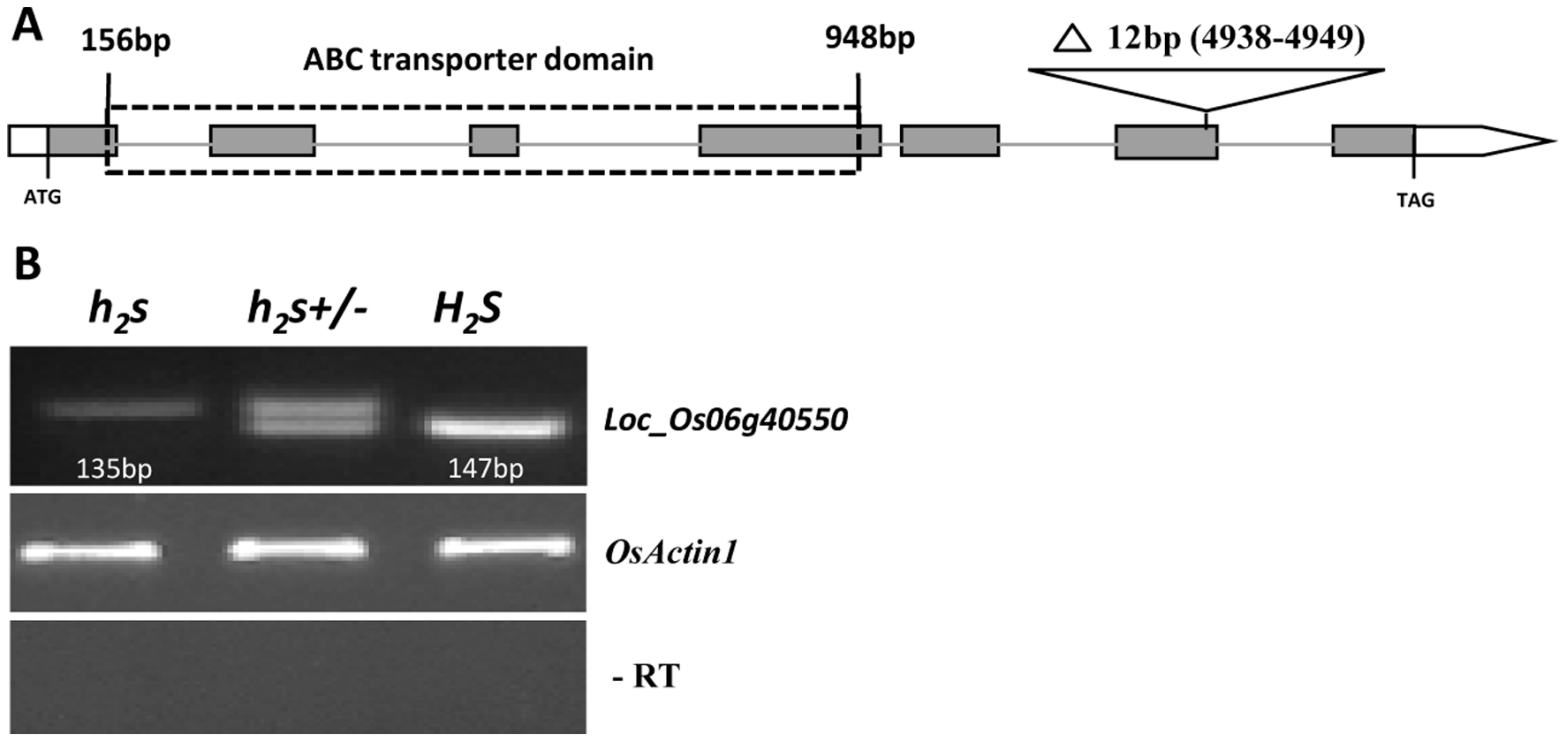
Sequencing analysis of candidate gene *Loc_Os06g40550.* (A) Schematic structure of gene *Loc_Os06g40550*, 12 bp deletion was detected at the predicted sixth exon. White and grey boxes for UTR regions and exons, respectively. The gray lines for introns, and the black dash box for ABC transporter domain region. (B) RT-PCR examination of 12 deletion in *Loc_Os06g40550* RNA. The deletion was detected in the RNA of heterozygous and homozygous plants.

A twelve-nucleotide (GCCACCCTCCTC) deletion in the predicted sixth exon of *Loc_Os06g40550* was detected in the *h_2_s* genomic DNA and RNA by sequencing and RT-PCR analysis (Figure5A and B). However, the deletion did not change the reading frame. These results suggest that the deletion in *Loc_Os06g40550* may be responsible for the *h_2_s* phenotype, but a complementary experiment is needed.

## Discussion

In CMS, cytoplasmic effects on most of the agronomic traits are either negative or not actively favorable to the achievement of high grain yield. Our study showed that the cytoplasmic effects of *h_2_s* on yield and GCA of yield were positive and significant in both years, suggesting that *h_2_s* may be more useful in improving rice yield than CMS. GMS does not have male sterile cytoplasm which is responsible for negative cytoplasmic effect on agronomic traits in the CMS [1], [5], [31]. For this reason, the cytoplasmic effects are probably positive in the GMS as *h_2_s*. The positive-cytoplasmic effects on yield in the GMS should be seriously considered in rice breeding.

Although the GMS mutant *h_2_s* shows promise for rice breeding, the challenge of maintaining the MS of *h_2_s* for the production of hybrid seeds limits its practical application. In order to utilize the positive-cytoplasmic effects of *h_2_s* and resolve the challenge, we could generate a PTMS line with both *h_2_s* cytoplasm and PTMS nuclei using nuclear substitutions. However, the fact that the fertility of PTMS line with *h_2_s* cytoplasm is easily affected by environment also restricts the wide application of positive cytoplasmic effects of *h_2_s*. The approach described by Marc et al. [7] has been proposed to maintain the male sterile condition of recessive genic MS and provide an alternative way of utilizing the positive-cytoplasmic effects of *h_2_s* in rice breeding. This approach requires cloning the gene responsible for the *h_2_s* phenotype. Our mapping results showed that no gene in the fine-mapping region has been reported to be responsible for *h_2_s* phenotype, suggesting that *h_2_s* is a novel gene essential to male reproductive development. Sequencing and bioinformatics analysis of the candidate genes suggested that *Loc_Os06g40550*, encoding a protein with ABC transporter domain, was the best candidate. This lays a strong basis for cloning the *H_2_S* gene and for using the *h_2_s* mutant in rice breeding. Because the *h_2_s* mutant showed defective rice anther and pollen wall development, our work may also be helpful for understanding the molecular mechanisms underlying the development of these structures.

## Supporting Information

Figure S1 **Two representative results of checking A1–A6 background using ISSR primers and SSRs.**
PPTX

Figure S2 **Seed set rate and CV (coefficient of variation) of 30 combinations of 6 isonuclear alloplasmic lines (A1–A6) with 5 restorers (R1–R5) during both years.**
PPTX

Figure S3 **Mean grain number per panicle and CV (coefficient of variation) of 30 combinations of 6 isonuclear alloplasmic lines (A1–A6) with 5 restorers (R1–R5) during both years.**
PPTX

Figure S4 **Mean 1000-grain weight and CV (coefficient of variation) of 30 combinations of 6 isonuclear alloplasmic lines (A1–A6) with 5 restorers (R1–R5) during both years.**
PPTX

Figure S5 **Mean effective panicle number and CV (coefficient of variation) of 30 combinations of 6 isonuclear alloplasmic lines (A1–A6) with 5 restorers (R1–R5) during both years.**
PPTX

Figure S6 **Mean plant height and CV (coefficient of variation) of 30 combinations of 6 isonuclear alloplasmic lines (A1–A6) with 5 restorers (R1–R5) during both years.**
PPTX

Figure S7 **Mean yield and CV (coefficient of variation) of 30 combinations of 6 isonuclear alloplasmic lines (A1–A6) with 5 restorers (R1–R5) during both years.**
PPTX

Figure S8 **Mean weight per panicle and CV (coefficient of variation) of 30 combinations of 6 isonuclear alloplasmic lines (A1–A6) with 5 restorers (R1–R5) during both years.**
PPTX

Table S1 **Isonuclear alloplasmic lines used in cytoplasm effects analysis.**
(DOCX)

Table S2 ***h_2_s*** **cytoplasmic effects on seed set rate, weight/panicle and yield in comparison with Zhenshan97A (A2), D702A (A3), G46A (A4), K18A (A5) and XieqingzaoA (A6).**
(DOCX)

Table S3 **GCA effects on seed set rate, weight per panicle and yield among cytoplasms of** ***h_2_s*** **(A1), Zhenshan97A (A2), D702A (A3), G46A (A4), K18A (A5) and XieqingzaoA (A6).**
(DOCX)

## References

[1] Chandra-Shekara AC, Prasanna BM, Singh BB, Unnikrishnan KV, Seetharam A (2006) Effect of cytoplasm and cytoplasm-nuclear interaction on combining ability and heterosis for agronomic traits in pearl millet {*Pennisetum glaucum* (L) Br. R}. Euphytica 153: 15–26.

[2] Tao D, Xu P, Zhou J, Deng X, Li J, et al (2011) Cytoplasm affects grain weight and filled-grain ratio in indica rice. BMC Genetics 12: 53. 21631950 10.1186/1471-2156-12-53 PMC3118132

[3] Shi C, Zhu J (1998) Genetic analysis of cytoplasmic and maternal effects for millling quality traits in indica rice. Seed Sci & Technol 26: 481–488.

[4] Young JB, Virmani SS (1990) Effects of cytoplasm on heterosis and combining ability for agronomic traits. Euphytica 48: 177–188.

[5] Wang W, Zhou K, Wen H, Zheng J, Zhu Y, et al (1997) The diversity of cytoplasmic effects on some quantitative traits in hybrid rice. Chinese J Rice Sci 11: 65–69.

[6] Yang S, Chen B, Shen W, Xia J (2009) Progress of application and breeding on two-line hybrid rice in China. Hybrid Rice 24: 5–9.

[7] Marc A, Timothy F, Howard H, Gary H, Yongzhong W (2006) Nucleotide sequences mediating plant male sterility and method of using same. International Patent International application number: PCT/US2006/024273.

[8] Ding J, Lu Q, Ouyang Y, Mao H, Zhang P, et al (2012) A long noncoding RNA regulates photoperiod-sensitive male sterility, an essential component of hybrid rice. Proc Natl Acad Sci USA 109: 2654–2659. 22308482 10.1073/pnas.1121374109 PMC3289353

[9] Zhou H, Liu Q, Li J, Jiang D, Zhou L, et al.. (2012) Photoperiod- and thermo-sensitive genic male sterility in rice are caused by a point mutation in a novel noncoding RNA that produces a small RNA. Cell Res. 10.1038/cr.2012.28 PMC3317565 22349461

[10] Jung KH, Han MJ, Lee Dy, Lee YS, Schreiber L, et al (2006) Wax-deficient anther1 is involved in cuticle and wax production in rice anther walls and is required for pollen development. Plant Cell 18: 3015–3032. 17138699 10.1105/tpc.106.042044 PMC1693940

[11] Li H, Pinot F, Sauveplane V, Werck-Reichhart D, Diehl P, et al (2010) Cytochrome P450 family member CYP704B2 catalyzes the hydroxylation of fatty acids and is required for anther cutin biosynthesis and pollen exine formation in rice. Plant Cell 22: 173–190. 20086189 10.1105/tpc.109.070326 PMC2828706

[12] Li N, Zhang DS, Liu HS, Yin CS, Li Xx, et al (2006) The rice tapetum degeneration retardation gene is required for tapetum degradation and anther development. Plant Cell 18: 2999–3014. 17138695 10.1105/tpc.106.044107 PMC1693939

[13] Aya K, Ueguchi-Tanaka M, Kondo M, Hamada K, Yano K, et al (2009) Gibberellin modulates anther development in rice via the transcriptional regulation of GAMYB. Plant Cell 21: 1453–1472. 19454733 10.1105/tpc.108.062935 PMC2700530

[14] Liu Z, Bao W, Liang W, Yin J, Zhang D (2010) Identification of gamyb-4 and analysis of the regulatory role of GAMYB in rice anther development. J Integr Plant Biol 52: 670–678. 20590996 10.1111/j.1744-7909.2010.00959.x

[15] Shi J, Tan H, Yu XH, Liu Y, Liang W, et al (2011) Defective pollen wall is required for anther and microspore development in rice and encodes a fatty acyl carrier protein reductase. Plant Cell 23: 2225–2246. 21705642 10.1105/tpc.111.087528 PMC3160036

[16] Li H, Yuan Z, Vizcay-Barrena G, Yang C, Liang W, et al (2011) PERSISTENT TAPETAL CELL1 encodes a PHD-Finger protein that is required for tapetal cell death and pollen development in rice. Plant Physiol 156: 615–630. 21515697 10.1104/pp.111.175760 PMC3177263

[17] Chen C, Xu Y, Ma H, Chong K (2005) Cell biological characterization of male meiosis and pollen development in rice. J Integr Plant Biol 47: 734–744.

[18] Feng J, Lu Y, Lu X, Xue X (2001) Pollen development and its stages in rice (*Oryza sativa L.*) Chinese J Rice Sci. 15: 21–28.

[19] Chen JB, He F, Qin P, Wang YP, Xu J, et al (2009) Genetic analysis and gene mapping of a rice recessive male sterile mutant. Plant Breeding 129: 313–317.

[20] Lander ES, Green P, Abrahamson J, Barlow A, Daly MJ, et al.. (1987) Mapmaker: an interactive computer package for constructin primary genetic linkage maps of experimental and natural populations. Genomics 1. 10.1016/0888-7543(87)90010-3 3692487

[21] Huang MD, Wei FJ, Wu CC, Hsing YIC, Huang AHC (2008) Analyses of advanced rice anther transcriptomes reveal global tapetum secretory functions and potential proteins for lipid exine formation. Plant Physiol 149: 694–707. 19091874 10.1104/pp.108.131128 PMC2633857

[22] Ouyang S, Zhu W, Hamilton J, Lin H, Campbell M, et al (2007) The TIGR Rice Genome Annotation Resource: improvements and new features. Nucleic Acids Res 35: D883–887. 17145706 10.1093/nar/gkl976 PMC1751532

[23] Baxter I, Vinegar B, Winter D, Nahal H, Ammar R, et al (2007) An “Electronic Fluorescent Pictograph” Browser for Exploring and Analyzing Large-Scale Biological Data Sets. PLoS ONE 2: e718. 17684564 10.1371/journal.pone.0000718 PMC1934936

[24] Lopez-Casado G, Urbanowicz BR, Damasceno CM, Rose JK (2008) Plant glycosyl hydrolases and biofuels: a natural marriage. Curr Opin Plant Biol 11: 329–337. 18396092 10.1016/j.pbi.2008.02.010

[25] Choi H, Jin J-Y, Choi S, Hwang J-U, Kim Y-Y, et al (2011) An ABCG/WBC-type ABC transporter is essential for transport of sporopollenin precursors for exine formation in developing pollen. Plant J 65: 181–193. 21223384 10.1111/j.1365-313X.2010.04412.x

[26] Quilichini TD, Friedmann MC, Samuels AL, Douglas CJ (2010) ATP-Binding Cassette Transporter G26 is required for male fertility and pollen exine formation in *Arabidopsis* . Plant Physiol 154: 678–690. 20732973 10.1104/pp.110.161968 PMC2949020

[27] Dou X, Yang K, Zhang Y, Wang W, Liu X, et al (2011) WBC27, an Adenosine Tri-phosphate-binding Cassette protein, controls pollen wall formation and patterning in *Arabidopsis* . J Integr Plant Biol 53: 74–88. 21205178 10.1111/j.1744-7909.2010.01010.x

[28] Ibl V, Csaszar E, Schlager N, Neubert S, Spitzer C, et al (2012) Interactome of the plant-specific ESCRT-III component AtVPS2.2 in *Arabidopsis thaliana* . J Proteome Res 11: 397–411. 22010978 10.1021/pr200845n PMC3252797

[29] Conchon S, Cao X, Barlowe C, Pelham HR (1999) Got1p and Sft2p: membrane proteins involved in traffic to the Golgi complex. EMBO J 18: 3934–3946. 10406798 10.1093/emboj/18.14.3934 PMC1171469

[30] Cheng MC, Hsieh EJ, Chen JH, Chen HY, Lin TP (2012) *Arabidopsis* RGLG2, functioning as a RING E3 ligase, interacts with AtERF53 and negatively regulates the plant drought stress response. Plant Physiol 158: 363–375. 22095047 10.1104/pp.111.189738 PMC3252077

[31] Tao D, Hu F, Yang J, Yang G, Yang Y, et al (2004) Cytoplasm and cytoplasm-nucleus interaction affect agronomic traits in *japonica* rice. Euphytica 135: 129–134.

