# Correction: Analysis of Cytoplasmic Effects and Fine-Mapping of a Genic Male Sterile Line in Rice

**DOI:** 10.1371/journal.pone.0156470

**Published:** 2016-05-23

**Authors:** Peng Qin, Yuping Wang, Yuanyuan Li, Bingtian Ma, Shigui Li, The PLOS ONE Staff

Following publication, it has come to the attention of the PLOS ONE Editors that this article contains substantial overlap with the following publication in Plant & Cell Physiology by some of the authors:

Qin, P., Tu, B., Wang, Y., Deng, L., Quilichini, T.D., Li, T., Wang, H., Ma, B. and Li, S. (2013) ABCG15 encodes an ABC transporter protein, and is essential for post-meiotic anther and pollen exine development in rice. Plant Cell Physiol, 54, 138–154. [2]

Specifically, the PLOS ONE paper reported findings related to phenotypic characterization of the male sterile mutant and the mapping of the mutation, which had previously been reported in [2]; this previous paper was not cited. Both papers were under editorial consideration during an overlapping time period.

The authors would like to clarify that the PLOS ONE article [1] mainly focused on cytoplasm effects and fine-mapping, while the Plant & Cell Physiology article [2] focused on ABCG15 cloning and functional analysis, and also included phenotype verification using different approaches.

The PLOS ONE Editors note that the failure by the authors to declare related work under consideration elsewhere is in breach of the journal’s editorial policies, and feel that combining the two reports into a single paper should have been considered.

The authors would like to apologize for the reporting of overlapping findings and the failure to cite the previous publication. Additionally, due to an error in figure preparation, the image used to create Fig 1E in the submission to PLOS ONE is a duplicate of the image used to create Fig 1D for the Plant & Cell Physiology article. Here we provide a corrected Fig 1.

**Fig 1:**
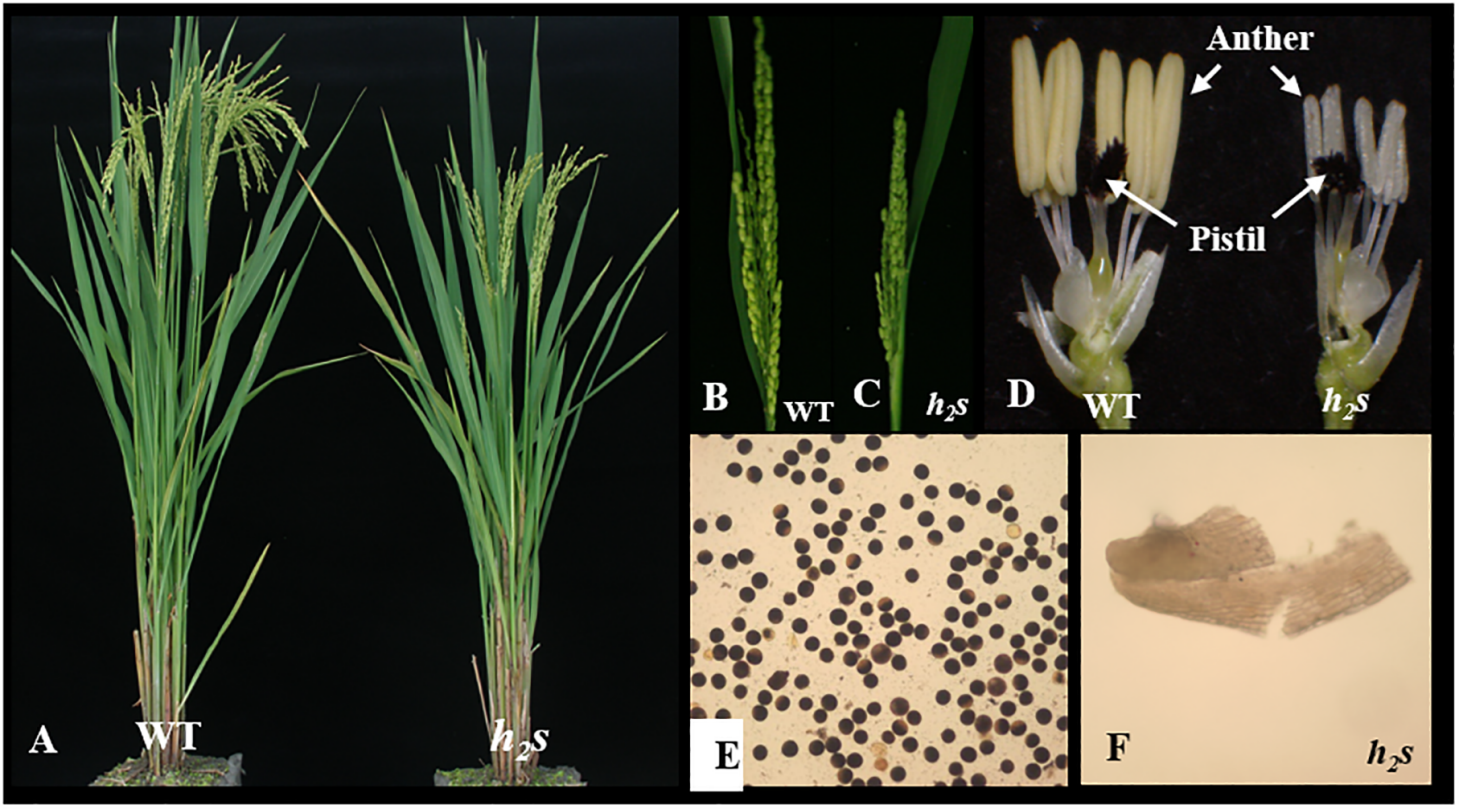
Phenotypic comparison of wild type (WT) and h_2_s. Comparison between WT (left) and h_2_s (right) for heading plant (A), headed panicle (B, C), flower (D) and pollen of WT (E) and anther of h_2_s (F).
