# Supplementary material for: Analysis of Cytoplasmic Effects and Fine-Mapping of a Genic Male Sterile Line in Rice
Source: PLoS One. 2013 Apr 16;8(4):e61719. doi: 10.1371/journal.pone.0061719 (PMC3628577; doi:10.1371/journal.pone.0061719)
Supplement: Figure S1 — Two representative results of checking A1–A6 background using ISSR primers and SSRs. PPTX [file pone.0061719.s001.pptx]

## Slide 1
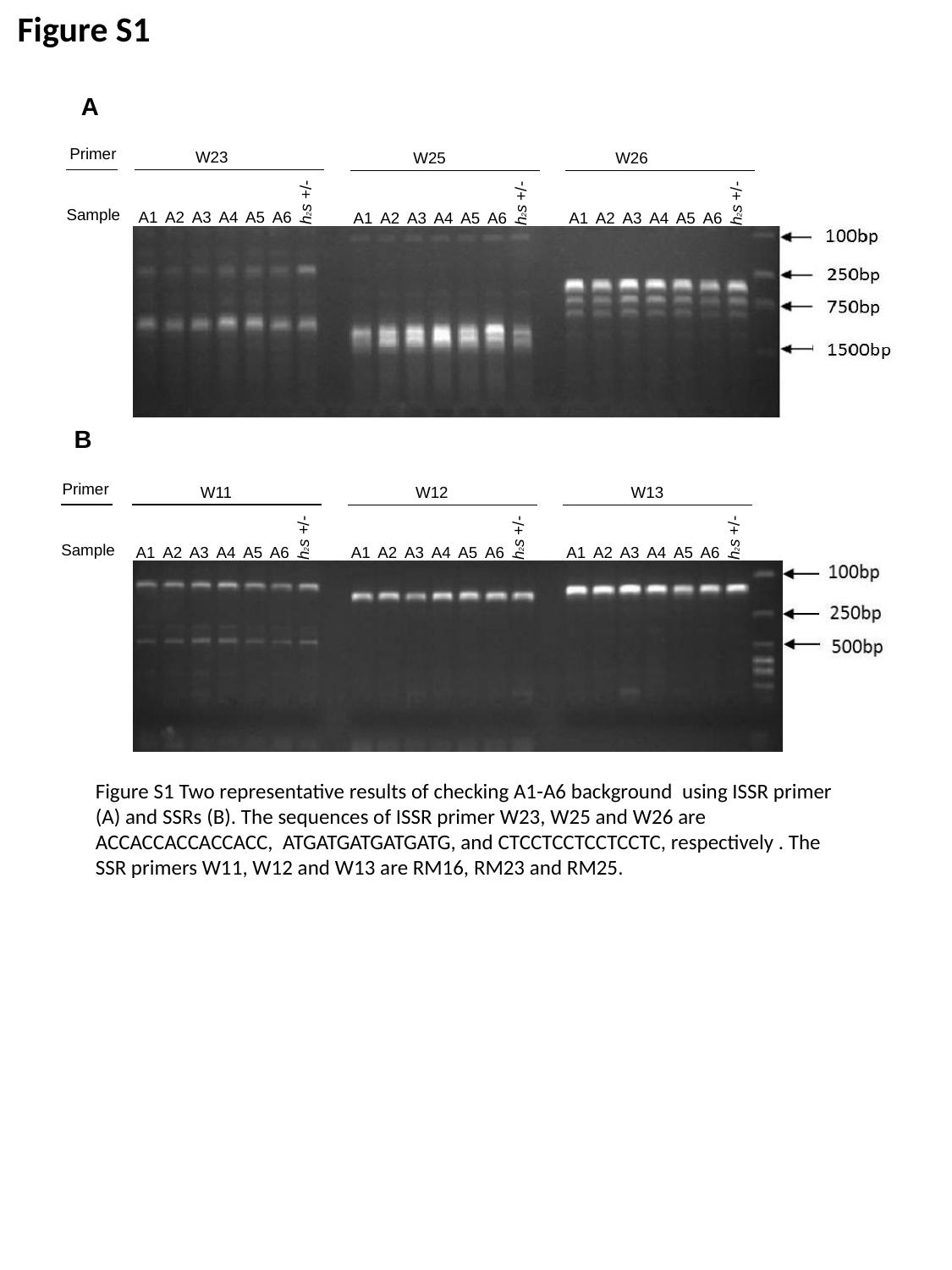

Figure S1
A
Primer
W23
W25
W26
h2s +/-
h2s +/-
h2s +/-
Sample
A1
A2
A3
A4
A5
A6
A1
A2
A3
A4
A5
A6
A1
A2
A3
A4
A5
A6
B
Primer
W11
W12
W13
h2s +/-
h2s +/-
h2s +/-
Sample
A1
A2
A3
A4
A5
A6
A1
A2
A3
A4
A5
A6
A1
A2
A3
A4
A5
A6
Figure S1 Two representative results of checking A1-A6 background using ISSR primer (A) and SSRs (B). The sequences of ISSR primer W23, W25 and W26 are ACCACCACCACCACC, ATGATGATGATGATG, and CTCCTCCTCCTCCTC, respectively . The SSR primers W11, W12 and W13 are RM16, RM23 and RM25.
