# Supplementary material for: Analysis of Cytoplasmic Effects and Fine-Mapping of a Genic Male Sterile Line in Rice
Source: PLoS One. 2013 Apr 16;8(4):e61719. doi: 10.1371/journal.pone.0061719 (PMC3628577; doi:10.1371/journal.pone.0061719)
Supplement: Figure S2 — Seed set rate and CV (coefficient of variation) of 30 combinations of 6 isonuclear alloplasmic lines (A1–A6) with 5 restorers (R1–R5) during both years. PPTX [file pone.0061719.s002.pptx]

## Slide 1
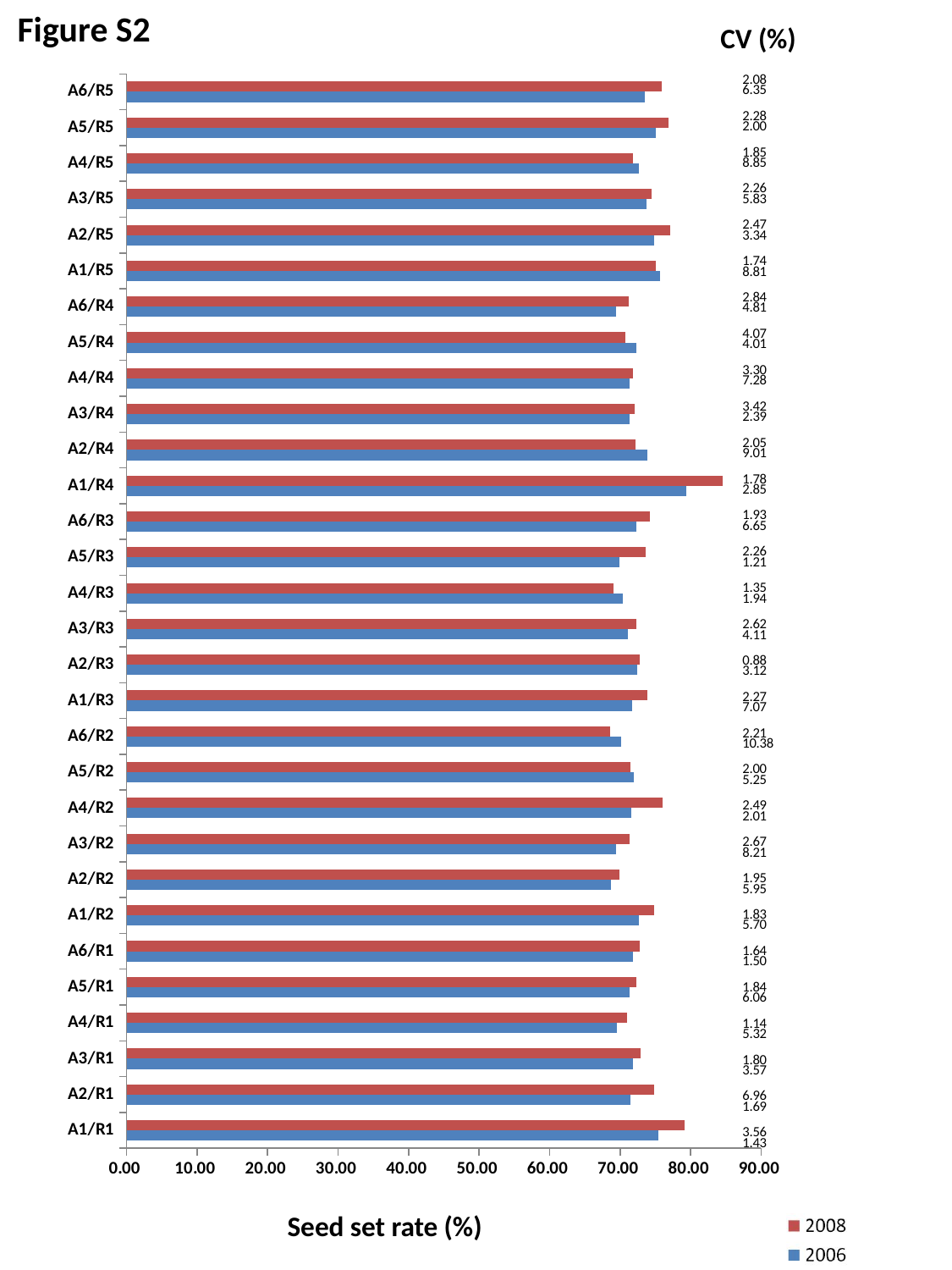

Figure S2
CV (%)
### Chart
| Category | | |
|---|---|---|
| A1/R1 | 75.43439678693294 | 79.09451425986937 |
| A2/R1 | 71.49449213961226 | 74.84952351541882 |
| A3/R1 | 71.79910356662155 | 72.90897224253155 |
| A4/R1 | 69.52730279814874 | 70.9425352631867 |
| A5/R1 | 71.37954614369765 | 72.27154578515331 |
| A6/R1 | 71.82049244133759 | 72.74821279045874 |
| A1/R2 | 72.69387877410423 | 74.86658453208703 |
| A2/R2 | 68.75303793544036 | 69.85873433715365 |
| A3/R2 | 69.43965722761733 | 71.32202985933864 |
| A4/R2 | 71.62567996368928 | 76.08157463652758 |
| A5/R2 | 71.93039525282836 | 71.50661862254275 |
| A6/R2 | 70.1787817822312 | 68.57700295233359 |
| A1/R3 | 71.69048393985771 | 73.85939342944066 |
| A2/R3 | 72.49187703630827 | 72.83870917315475 |
| A3/R3 | 71.10716519416563 | 72.27494134114586 |
| A4/R3 | 70.39979641125011 | 69.0884195386789 |
| A5/R3 | 69.93368758723442 | 73.6050562820623 |
| A6/R3 | 72.36538683589815 | 74.25888967707411 |
| A1/R4 | 79.38697260337061 | 84.61125317105075 |
| A2/R4 | 73.85035996494203 | 72.1937710486518 |
| A3/R4 | 71.38666044735857 | 72.09957299235694 |
| A4/R4 | 71.32180760936207 | 71.83907658877816 |
| A5/R4 | 72.37246205834377 | 70.78834697052335 |
| A6/R4 | 69.37852427370727 | 71.19726944063659 |
| A1/R5 | 75.7292830669816 | 75.02110464942969 |
| A2/R5 | 74.85110095728363 | 77.1082873743122 |
| A3/R5 | 73.7248647453467 | 74.44814476408243 |
| A4/R5 | 72.67378048418796 | 71.79943611902607 |
| A5/R5 | 75.03902917496403 | 76.9204214551281 |
| A6/R5 | 73.53012047160259 | 75.90446014518777 |2.08
6.35
2.28
2.00
1.85
8.85
2.26
5.83
2.47
3.34
1.74
8.81
2.84
4.81
4.07
4.01
3.30
7.28
3.42
2.39
2.05
9.01
1.78
2.85
1.93
6.65
2.26
1.21
1.35
1.94
2.62
4.11
0.88
3.12
2.27
7.07
2.21
10.38
2.00
5.25
2.49
2.01
2.67
8.21
1.95
5.95
1.83
5.70
1.64
1.50
1.84
6.06
1.14
5.32
1.80
3.57
6.96
1.69
3.56
1.43
Seed set rate (%)
