# Supplementary material for: Analysis of Cytoplasmic Effects and Fine-Mapping of a Genic Male Sterile Line in Rice
Source: PLoS One. 2013 Apr 16;8(4):e61719. doi: 10.1371/journal.pone.0061719 (PMC3628577; doi:10.1371/journal.pone.0061719)
Supplement: Figure S3 — Mean grain number per panicle and CV (coefficient of variation) of 30 combinations of 6 isonuclear alloplasmic lines (A1–A6) with 5 restorers (R1–R5) during both years. PPTX [file pone.0061719.s003.pptx]

## Slide 1
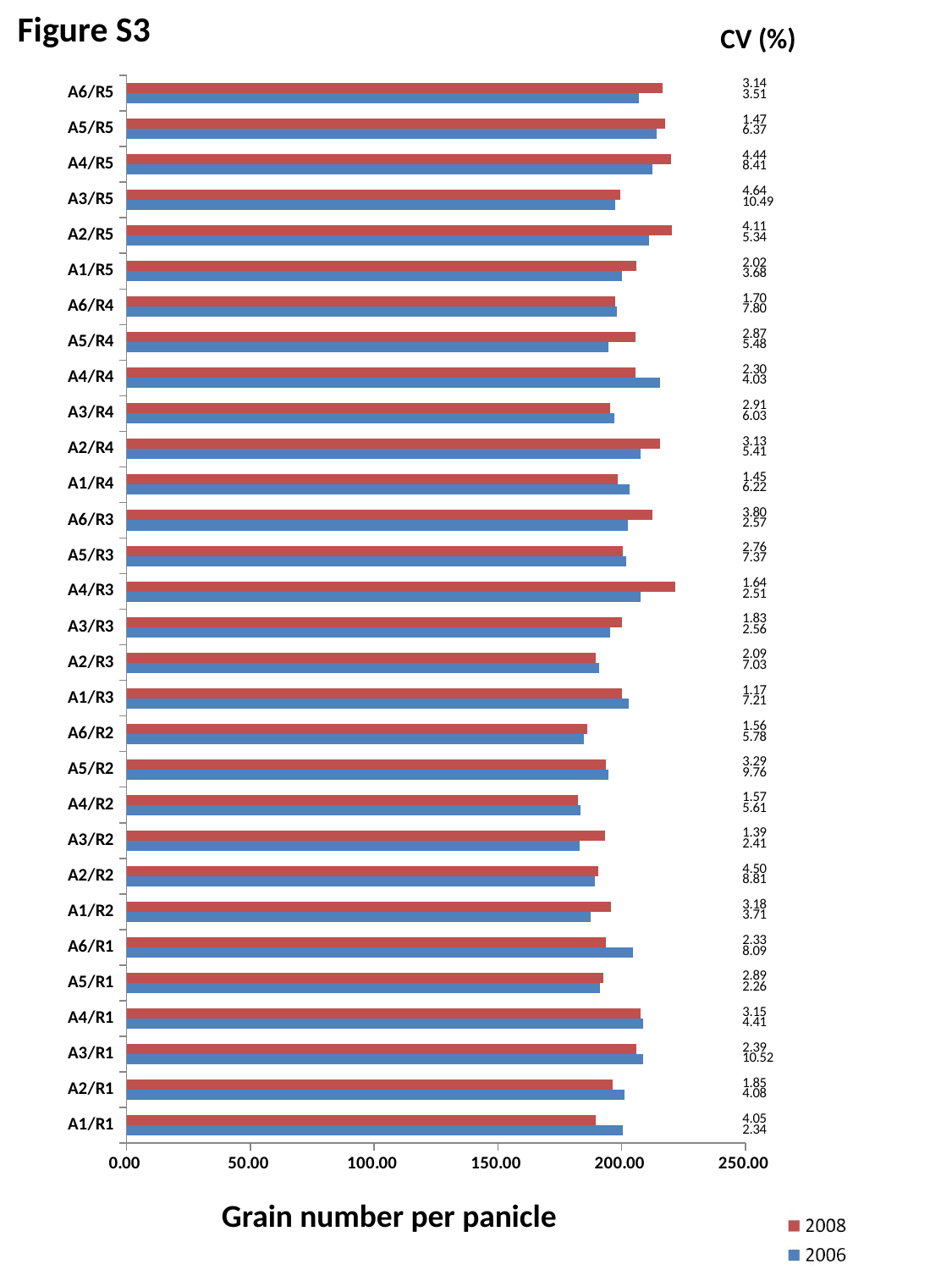

Figure S3
CV (%)
### Chart
| Category | | |
|---|---|---|
| A1/R1 | 200.54152937646782 | 189.59956722449658 |
| A2/R1 | 201.09924078526265 | 196.299962952896 |
| A3/R1 | 208.73528379304935 | 205.83150267661395 |
| A4/R1 | 208.6524625596584 | 207.47714053270374 |
| A5/R1 | 191.29169634245747 | 192.48830092431157 |
| A6/R1 | 204.6317436843914 | 193.72088529378 |
| A1/R2 | 187.49953030884967 | 195.72186634776085 |
| A2/R2 | 189.0111072385886 | 190.55372864657772 |
| A3/R2 | 182.99359889963566 | 193.32049266053687 |
| A4/R2 | 183.31591695501734 | 182.1576374908807 |
| A5/R2 | 194.6914214754237 | 193.75374220146895 |
| A6/R2 | 184.74957506327283 | 186.21383941638098 |
| A1/R3 | 202.88990977209582 | 200.01105787241178 |
| A2/R3 | 190.96238818113522 | 189.6341956276178 |
| A3/R3 | 195.39647146200332 | 200.22335906875585 |
| A4/R3 | 207.72456506907722 | 221.52386078902305 |
| A5/R3 | 201.71446728784565 | 200.56549951314705 |
| A6/R3 | 202.41473002356318 | 212.54878006613754 |
| A1/R4 | 203.11505140123052 | 198.36355905828086 |
| A2/R4 | 207.53420541854493 | 215.34407275211274 |
| A3/R4 | 197.13016588118333 | 195.36964187693874 |
| A4/R4 | 215.60218161257936 | 205.6208304767886 |
| A5/R4 | 194.59928140997147 | 205.45796415229708 |
| A6/R4 | 197.96450391793482 | 197.5152188573409 |
| A1/R5 | 199.93167852607385 | 205.96750594729352 |
| A2/R5 | 211.14578144959765 | 220.37148477028907 |
| A3/R5 | 197.31580556625633 | 199.32444324412532 |
| A4/R5 | 212.4945940461656 | 219.8444931730339 |
| A5/R5 | 214.02200626216464 | 217.3842110181448 |
| A6/R5 | 206.85276851811622 | 216.33872714408622 |3.14
3.51
1.47
6.37
4.44
8.41
4.64
10.49
4.11
5.34
2.02
3.68
1.70
7.80
2.87
5.48
2.30
4.03
2.91
6.03
3.13
5.41
1.45
6.22
3.80
2.57
2.76
7.37
1.64
2.51
1.83
2.56
2.09
7.03
1.17
7.21
1.56
5.78
3.29
9.76
1.57
5.61
1.39
2.41
4.50
8.81
3.18
3.71
2.33
8.09
2.89
2.26
3.15
4.41
2.39
10.52
1.85
4.08
4.05
2.34
Grain number per panicle
