# Supplementary material for: Analysis of Cytoplasmic Effects and Fine-Mapping of a Genic Male Sterile Line in Rice
Source: PLoS One. 2013 Apr 16;8(4):e61719. doi: 10.1371/journal.pone.0061719 (PMC3628577; doi:10.1371/journal.pone.0061719)
Supplement: Figure S4 — Mean 1000-grain weight and CV (coefficient of variation) of 30 combinations of 6 isonuclear alloplasmic lines (A1–A6) with 5 restorers (R1–R5) during both years. PPTX [file pone.0061719.s004.pptx]

## Slide 1
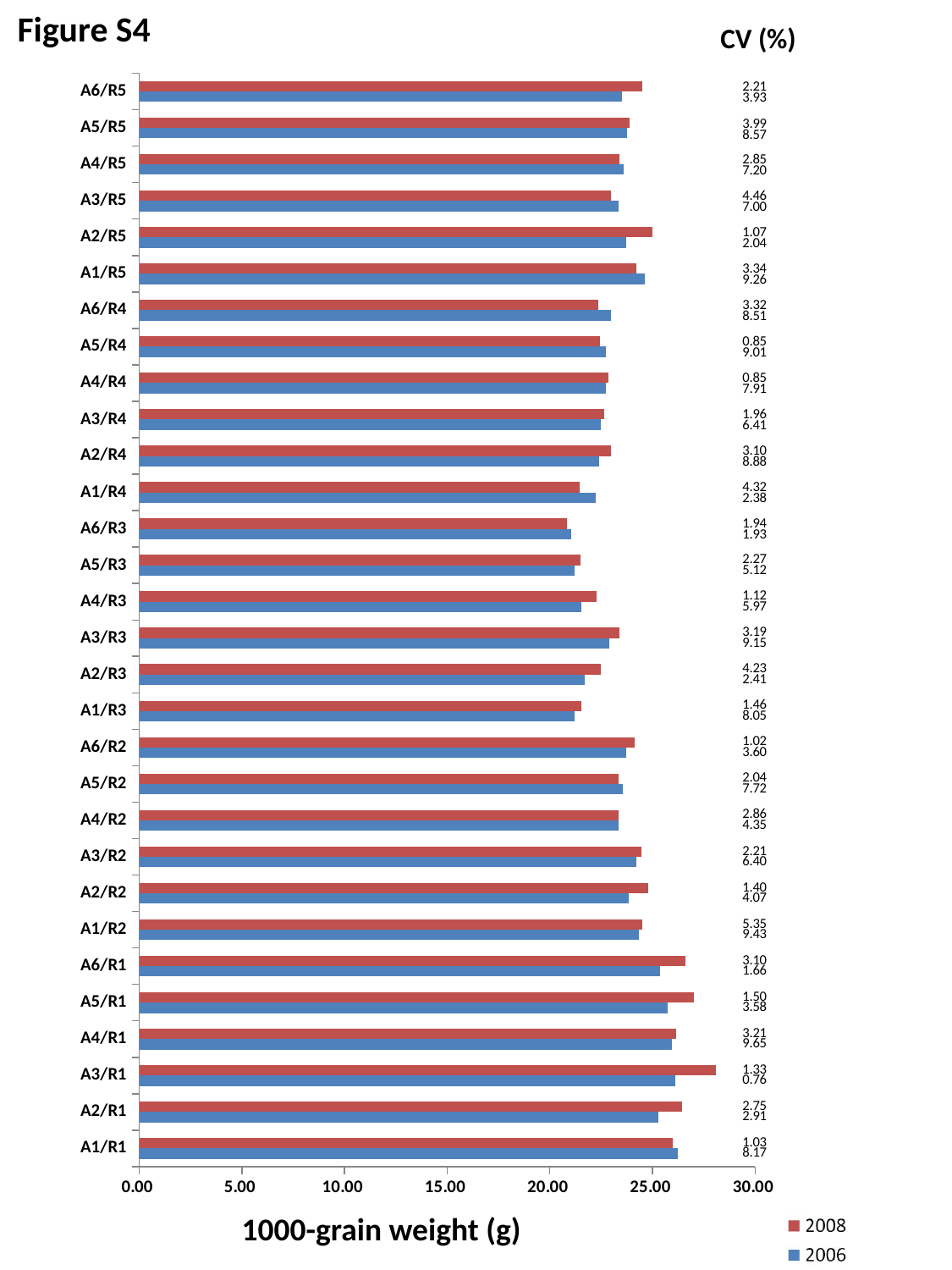

Figure S4
CV (%)
### Chart
| Category | | |
|---|---|---|
| A1/R1 | 26.257777777777765 | 25.978123602684054 |
| A2/R1 | 25.277777777777775 | 26.44892077844365 |
| A3/R1 | 26.11111111111111 | 28.107337084345122 |
| A4/R1 | 25.955555555555545 | 26.13580523823437 |
| A5/R1 | 25.722222222222225 | 27.00066678900458 |
| A6/R1 | 25.38888888888889 | 26.588830797596344 |
| A1/R2 | 24.333333333333332 | 24.50361557272006 |
| A2/R2 | 23.833333333333332 | 24.81437416321583 |
| A3/R2 | 24.222222222222218 | 24.46573191298604 |
| A4/R2 | 23.333333333333332 | 23.35718461654636 |
| A5/R2 | 23.555555555555554 | 23.35737793930637 |
| A6/R2 | 23.71111111111111 | 24.15179369853244 |
| A1/R3 | 21.188888888888886 | 21.52694652021583 |
| A2/R3 | 21.722222222222232 | 22.471843678008018 |
| A3/R3 | 22.9111111111111 | 23.40854823021898 |
| A4/R3 | 21.527777777777782 | 22.275861929765707 |
| A5/R3 | 21.22222222222222 | 21.500442779530392 |
| A6/R3 | 21.048888888888886 | 20.85101919108473 |
| A1/R4 | 22.233333333333324 | 21.448102219377684 |
| A2/R4 | 22.38888888888889 | 22.976642470522936 |
| A3/R4 | 22.5 | 22.636140634412858 |
| A4/R4 | 22.722222222222218 | 22.848424596502 |
| A5/R4 | 22.722222222222218 | 22.44201324897 |
| A6/R4 | 23.0 | 22.3525336283929 |
| A1/R5 | 24.611111111111114 | 24.238199442973023 |
| A2/R5 | 23.722222222222225 | 25.008787281442736 |
| A3/R5 | 23.333333333333346 | 23.0012449772307 |
| A4/R5 | 23.61111111111111 | 23.373184388987024 |
| A5/R5 | 23.777777777777775 | 23.870948055471846 |
| A6/R5 | 23.533333333333335 | 24.515478064967315 |2.21
3.93
3.99
8.57
2.85
7.20
4.46
7.00
1.07
2.04
3.34
9.26
3.32
8.51
0.85
9.01
0.85
7.91
1.96
6.41
3.10
8.88
4.32
2.38
1.94
1.93
2.27
5.12
1.12
5.97
3.19
9.15
4.23
2.41
1.46
8.05
1.02
3.60
2.04
7.72
2.86
4.35
2.21
6.40
1.40
4.07
5.35
9.43
3.10
1.66
1.50
3.58
3.21
9.65
1.33
0.76
2.75
2.91
1.03
8.17
1000-grain weight (g)
