# Supplementary material for: Analysis of Cytoplasmic Effects and Fine-Mapping of a Genic Male Sterile Line in Rice
Source: PLoS One. 2013 Apr 16;8(4):e61719. doi: 10.1371/journal.pone.0061719 (PMC3628577; doi:10.1371/journal.pone.0061719)
Supplement: Figure S5 — Mean effective panicle number and CV (coefficient of variation) of 30 combinations of 6 isonuclear alloplasmic lines (A1–A6) with 5 restorers (R1–R5) during both years. PPTX [file pone.0061719.s005.pptx]

## Slide 1
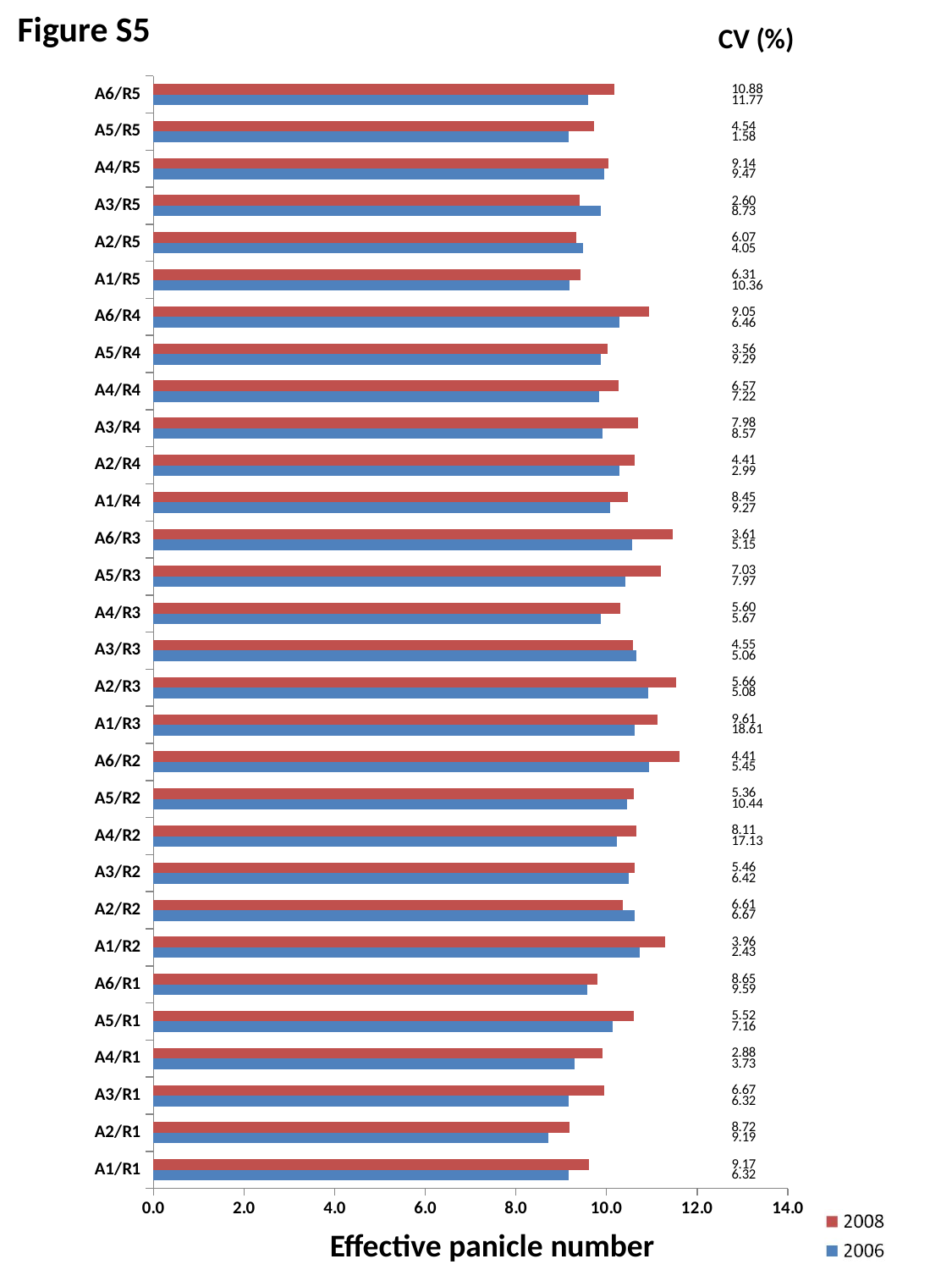

Figure S5
CV (%)
### Chart
| Category | | |
|---|---|---|
| A1/R1 | 9.166666666666666 | 9.620488079499845 |
| A2/R1 | 8.716666666666667 | 9.189771443207148 |
| A3/R1 | 9.166666666666666 | 9.947925491919547 |
| A4/R1 | 9.299999999999999 | 9.912202534322804 |
| A5/R1 | 10.133333333333333 | 10.61142971252423 |
| A6/R1 | 9.583333333333334 | 9.79256807761414 |
| A1/R2 | 10.733333333333334 | 11.299771799441714 |
| A2/R2 | 10.616666666666667 | 10.368702467224727 |
| A3/R2 | 10.483333333333333 | 10.620927547633721 |
| A4/R2 | 10.233333333333333 | 10.658886267318334 |
| A5/R2 | 10.450000000000001 | 10.598180380907797 |
| A6/R2 | 10.947407407407406 | 11.606210066841838 |
| A1/R3 | 10.613333333333333 | 11.12102953671412 |
| A2/R3 | 10.916666666666666 | 11.543550482096286 |
| A3/R3 | 10.666666666666666 | 10.59190478205016 |
| A4/R3 | 9.883333333333335 | 10.311133637852935 |
| A5/R3 | 10.416666666666666 | 11.191448524403967 |
| A6/R3 | 10.563333333333334 | 11.46580734507863 |
| A1/R4 | 10.083333333333334 | 10.468530696821102 |
| A2/R4 | 10.283333333333333 | 10.628770794120557 |
| A3/R4 | 9.916666666666666 | 10.703587731499555 |
| A4/R4 | 9.846296296296297 | 10.258543978225454 |
| A5/R4 | 9.866666666666665 | 10.022999268646226 |
| A6/R4 | 10.27962962962963 | 10.93130519110875 |
| A1/R5 | 9.183333333333335 | 9.431496501730319 |
| A2/R5 | 9.483333333333334 | 9.32573989774582 |
| A3/R5 | 9.883333333333333 | 9.406392513022295 |
| A4/R5 | 9.950000000000001 | 10.0463708633798 |
| A5/R5 | 9.166666666666666 | 9.719934202681626 |
| A6/R5 | 9.6 | 10.177410964688908 |10.88
11.77
4.54
1.58
9.14
9.47
2.60
8.73
6.07
4.05
6.31
10.36
9.05
6.46
3.56
9.29
6.57
7.22
7.98
8.57
4.41
2.99
8.45
9.27
3.61
5.15
7.03
7.97
5.60
5.67
4.55
5.06
5.66
5.08
9.61
18.61
4.41
5.45
5.36
10.44
8.11
17.13
5.46
6.42
6.61
6.67
3.96
2.43
8.65
9.59
5.52
7.16
2.88
3.73
6.67
6.32
8.72
9.19
9.17
6.32
Effective panicle number
