# Supplementary material for: Analysis of Cytoplasmic Effects and Fine-Mapping of a Genic Male Sterile Line in Rice
Source: PLoS One. 2013 Apr 16;8(4):e61719. doi: 10.1371/journal.pone.0061719 (PMC3628577; doi:10.1371/journal.pone.0061719)
Supplement: Figure S6 — Mean plant height and CV (coefficient of variation) of 30 combinations of 6 isonuclear alloplasmic lines (A1–A6) with 5 restorers (R1–R5) during both years. PPTX [file pone.0061719.s006.pptx]

## Slide 1
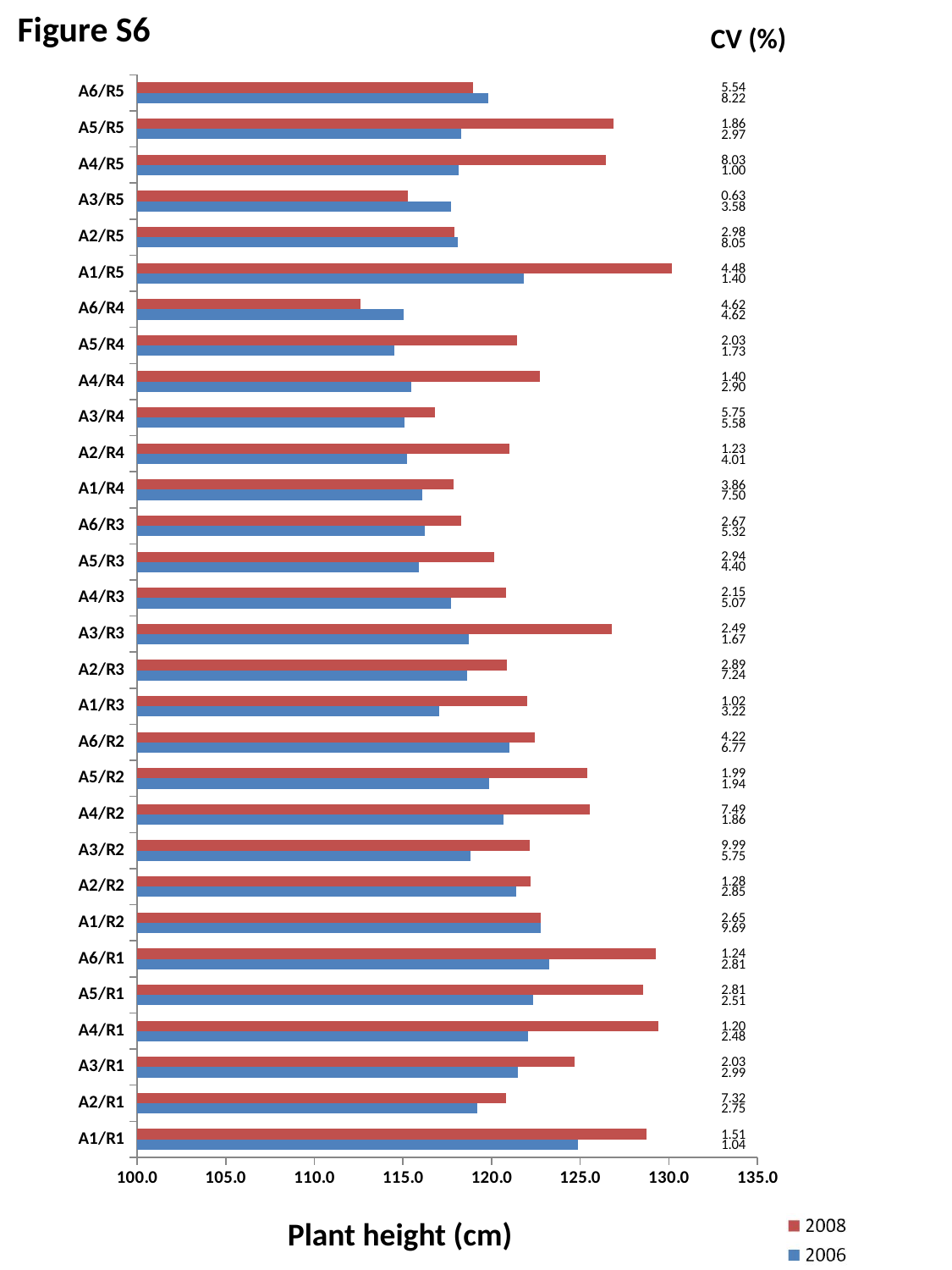

Figure S6
CV (%)
### Chart
| Category | | |
|---|---|---|
| A1/R1 | 124.86666666666667 | 128.72963044538702 |
| A2/R1 | 119.19999999999999 | 120.80697702940127 |
| A3/R1 | 121.46666666666665 | 124.66677993202607 |
| A4/R1 | 122.04074074074067 | 129.40223170642017 |
| A5/R1 | 122.36666666666667 | 128.56776333165053 |
| A6/R1 | 123.26666666666665 | 129.27209672987053 |
| A1/R2 | 122.77777777777777 | 122.78621203378884 |
| A2/R2 | 121.36666666666667 | 122.201378022891 |
| A3/R2 | 118.8 | 122.14432817969339 |
| A4/R2 | 120.66666666666667 | 125.534191666106 |
| A5/R2 | 119.86666666666667 | 125.411178019884 |
| A6/R2 | 121.01555555555568 | 122.44470506671276 |
| A1/R3 | 117.04 | 121.99887843820561 |
| A2/R3 | 118.63333333333333 | 120.85684532180484 |
| A3/R3 | 118.7 | 126.78032071313514 |
| A4/R3 | 117.7 | 120.82347314316688 |
| A5/R3 | 115.90000000000002 | 120.16420774957243 |
| A6/R3 | 116.23481481481467 | 118.26332565074125 |
| A1/R4 | 116.09999999999998 | 117.8717659533782 |
| A2/R4 | 115.23333333333333 | 121.01558721598606 |
| A3/R4 | 115.06666666666666 | 116.7848073839309 |
| A4/R4 | 115.44444444444444 | 122.7359811993067 |
| A5/R4 | 114.53333333333335 | 121.44514529830805 |
| A6/R4 | 115.04074074074067 | 112.6135952013558 |
| A1/R5 | 121.83333333333333 | 130.17495127341047 |
| A2/R5 | 118.10000000000001 | 117.89793350197488 |
| A3/R5 | 117.73333333333333 | 115.28637809714776 |
| A4/R5 | 118.16666666666667 | 126.43481639985576 |
| A5/R5 | 118.26666666666667 | 126.87043369926987 |
| A6/R5 | 119.80740740740741 | 118.97761721217182 |5.54
8.22
1.86
2.97
8.03
1.00
0.63
3.58
2.98
8.05
4.48
1.40
4.62
4.62
2.03
1.73
1.40
2.90
5.75
5.58
1.23
4.01
3.86
7.50
2.67
5.32
2.94
4.40
2.15
5.07
2.49
1.67
2.89
7.24
1.02
3.22
4.22
6.77
1.99
1.94
7.49
1.86
9.99
5.75
1.28
2.85
2.65
9.69
1.24
2.81
2.81
2.51
1.20
2.48
2.03
2.99
7.32
2.75
1.51
1.04
Plant height (cm)
