# Supplementary material for: Analysis of Cytoplasmic Effects and Fine-Mapping of a Genic Male Sterile Line in Rice
Source: PLoS One. 2013 Apr 16;8(4):e61719. doi: 10.1371/journal.pone.0061719 (PMC3628577; doi:10.1371/journal.pone.0061719)
Supplement: Figure S7 — Mean yield and CV (coefficient of variation) of 30 combinations of 6 isonuclear alloplasmic lines (A1–A6) with 5 restorers (R1–R5) during both years. PPTX [file pone.0061719.s007.pptx]

## Slide 1
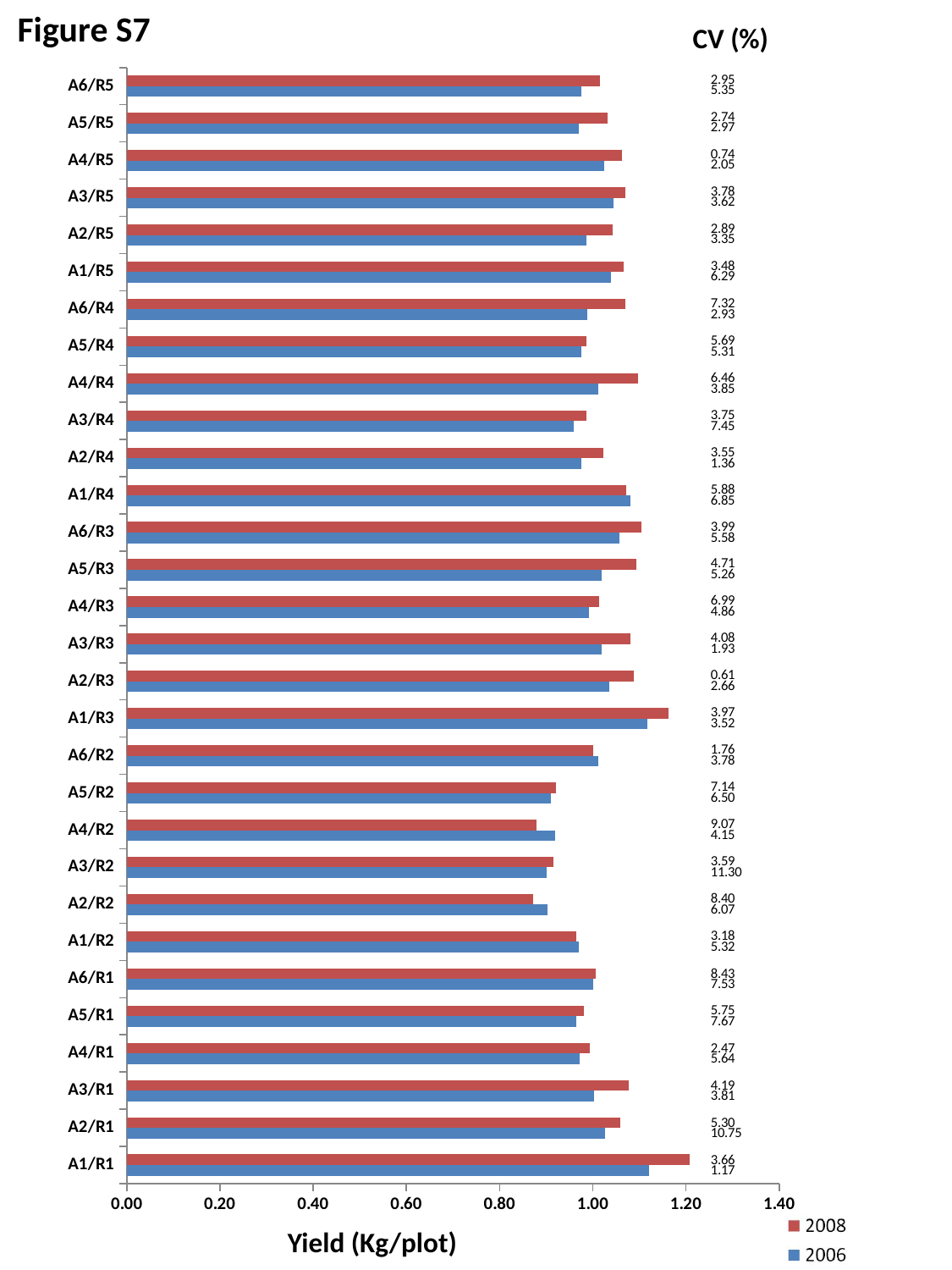

Figure S7
CV (%)
### Chart
| Category | | |
|---|---|---|
| A1/R1 | 1.1213333333333333 | 1.2082600135864354 |
| A2/R1 | 1.0266666666666666 | 1.0590935217341888 |
| A3/R1 | 1.002 | 1.0779099504371772 |
| A4/R1 | 0.972 | 0.993826104471003 |
| A5/R1 | 0.964 | 0.980692671211368 |
| A6/R1 | 1.0 | 1.006511559387489 |
| A1/R2 | 0.9700866666666667 | 0.9644766079862489 |
| A2/R2 | 0.9028866666666667 | 0.8719178702924136 |
| A3/R2 | 0.9013333333333334 | 0.9152086114118606 |
| A4/R2 | 0.9196666666666666 | 0.8802032153208202 |
| A5/R2 | 0.9106666666666666 | 0.9205939851833703 |
| A6/R2 | 1.0118219999999998 | 1.0009137646541093 |
| A1/R3 | 1.1164466666666666 | 1.1624014702021 |
| A2/R3 | 1.03562 | 1.0881050422610032 |
| A3/R3 | 1.02 | 1.0801273223611603 |
| A4/R3 | 0.9926666666666667 | 1.0128996088098419 |
| A5/R3 | 1.02 | 1.0936188945598149 |
| A6/R3 | 1.0563333333333333 | 1.1042170083331062 |
| A1/R4 | 1.08 | 1.0723720158959578 |
| A2/R4 | 0.976 | 1.0228465395348676 |
| A3/R4 | 0.96 | 0.9862402986138505 |
| A4/R4 | 1.0126733333333333 | 1.0964813523754302 |
| A5/R4 | 0.976 | 0.9863946394759621 |
| A6/R4 | 0.98834 | 1.0705500672524795 |
| A1/R5 | 1.0383333333333333 | 1.0671239553326923 |
| A2/R5 | 0.9869466666666667 | 1.0423350711234214 |
| A3/R5 | 1.0446 | 1.070327411641183 |
| A4/R5 | 1.0246666666666666 | 1.062379144263969 |
| A5/R5 | 0.9706666666666667 | 1.0322707763231762 |
| A6/R5 | 0.9749333333333333 | 1.015507729061193 |2.95
5.35
2.74
2.97
0.74
2.05
3.78
3.62
2.89
3.35
3.48
6.29
7.32
2.93
5.69
5.31
6.46
3.85
3.75
7.45
3.55
1.36
5.88
6.85
3.99
5.58
4.71
5.26
6.99
4.86
4.08
1.93
0.61
2.66
3.97
3.52
1.76
3.78
7.14
6.50
9.07
4.15
3.59
11.30
8.40
6.07
3.18
5.32
8.43
7.53
5.75
7.67
2.47
5.64
4.19
3.81
5.30
10.75
3.66
1.17
Yield (Kg/plot)
