# Supplementary material for: Analysis of Cytoplasmic Effects and Fine-Mapping of a Genic Male Sterile Line in Rice
Source: PLoS One. 2013 Apr 16;8(4):e61719. doi: 10.1371/journal.pone.0061719 (PMC3628577; doi:10.1371/journal.pone.0061719)
Supplement: Figure S8 — Mean weight per panicle and CV (coefficient of variation) of 30 combinations of 6 isonuclear alloplasmic lines (A1–A6) with 5 restorers (R1–R5) during both years. PPTX [file pone.0061719.s008.pptx]

## Slide 1
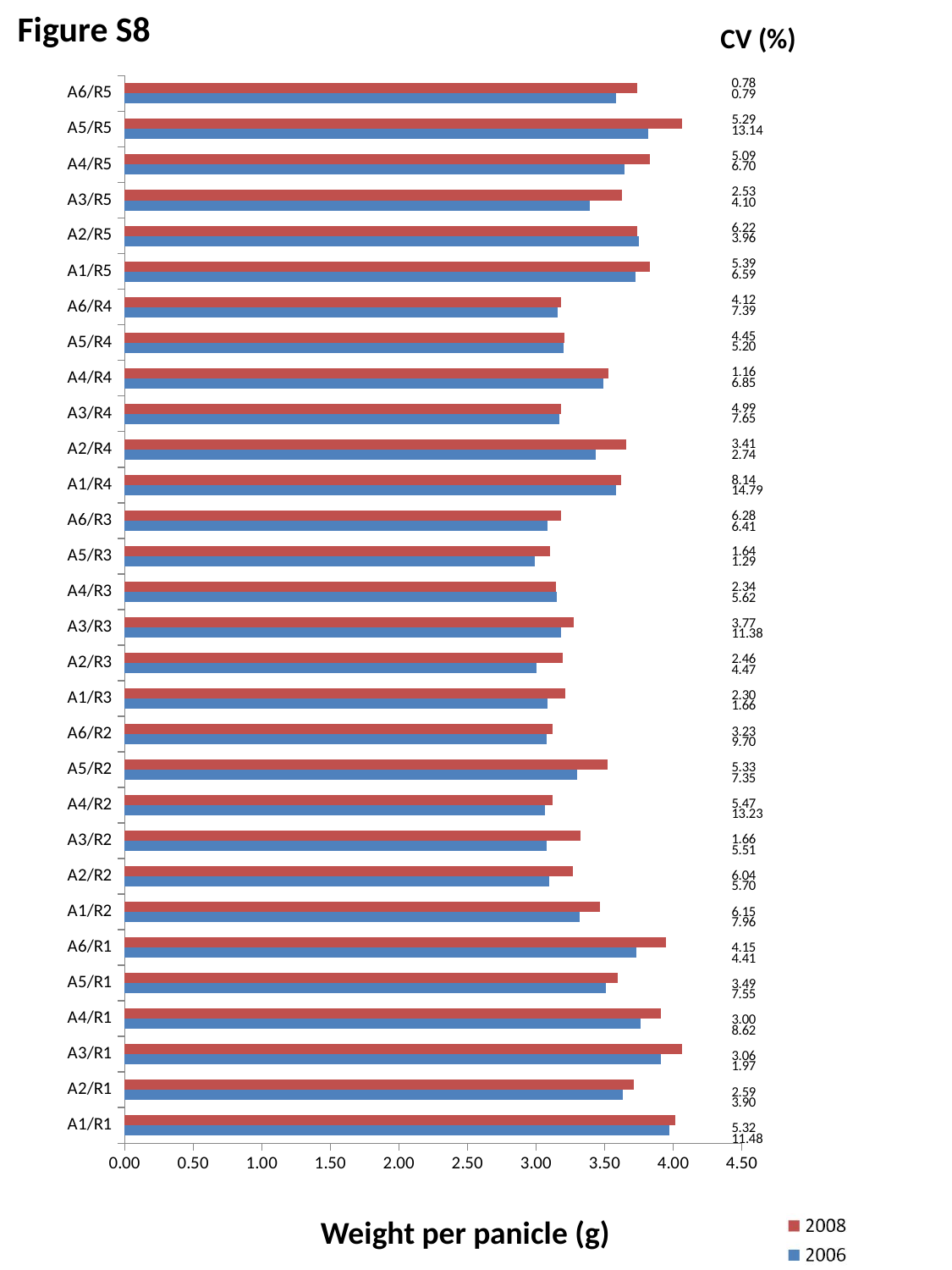

Figure S8
CV (%)
### Chart
| Category | | |
|---|---|---|
| A1/R1 | 3.973328069926699 | 4.0146933503258575 |
| A2/R1 | 3.633448612934909 | 3.7157916126638857 |
| A3/R1 | 3.9130890451610933 | 4.066308695476333 |
| A4/R1 | 3.7637957957935555 | 3.9112169884967636 |
| A5/R1 | 3.5119878418426644 | 3.5944593340793496 |
| A6/R1 | 3.7312364794579227 | 3.947392238111529 |
| A1/R2 | 3.316046771222076 | 3.4639471250468223 |
| A2/R2 | 3.0982841520581386 | 3.267260679887555 |
| A3/R2 | 3.0767827862831525 | 3.3237983716265598 |
| A4/R2 | 3.0649836601796365 | 3.119404092328331 |
| A5/R2 | 3.299744408109337 | 3.5218548087342576 |
| A6/R2 | 3.0744691692220756 | 3.1175499223525556 |
| A1/R3 | 3.0816410797746006 | 3.2103849226658485 |
| A2/R3 | 3.0053577087771104 | 3.193272662406219 |
| A3/R3 | 3.1826197683403863 | 3.274867346507144 |
| A4/R3 | 3.1481436888230365 | 3.141955765277165 |
| A5/R3 | 2.9922295740059863 | 3.101067300236291 |
| A6/R3 | 3.081015966931942 | 3.1826008566894313 |
| A1/R4 | 3.5855523601238333 | 3.6195916148872875 |
| A2/R4 | 3.433160402154128 | 3.656284017840975 |
| A3/R4 | 3.1706089424198134 | 3.182030161419545 |
| A4/R4 | 3.4934400552909004 | 3.529880183473884 |
| A5/R4 | 3.2000538234727407 | 3.2092765891524366 |
| A6/R4 | 3.1567625378396307 | 3.1843256960661415 |
| A1/R5 | 3.7264974042951615 | 3.8283883720926677 |
| A2/R5 | 3.748253457530423 | 3.737396834446456 |
| A3/R5 | 3.39240825610964 | 3.6268212456665148 |
| A4/R5 | 3.647076062956643 | 3.8291853819928163 |
| A5/R5 | 3.816576816163302 | 4.067068993622219 |
| A6/R5 | 3.5802523032031286 | 3.7383290405125753 |0.78
0.79
5.29
13.14
5.09
6.70
2.53
4.10
6.22
3.96
5.39
6.59
4.12
7.39
4.45
5.20
1.16
6.85
4.99
7.65
3.41
2.74
8.14
14.79
6.28
6.41
1.64
1.29
2.34
5.62
3.77
11.38
2.46
4.47
2.30
1.66
3.23
9.70
5.33
7.35
5.47
13.23
1.66
5.51
6.04
5.70
6.15
7.96
4.15
4.41
3.49
7.55
3.00
8.62
3.06
1.97
2.59
3.90
5.32
11.48
Weight per panicle (g)
