# Supplementary material for: Analysis of Cytoplasmic Effects and Fine-Mapping of a Genic Male Sterile Line in Rice
Source: PLoS One. 2013 Apr 16;8(4):e61719. doi: 10.1371/journal.pone.0061719 (PMC3628577; doi:10.1371/journal.pone.0061719)
Supplement: Table S1 — Isonuclear alloplasmic lines used in cytoplasm effects analysis. (DOCX) [file pone.0061719.s009.docx]

Table S1 Isonuclear alloplasmic lines used in cytoplasm effects analysis

| Experimental code | Nucleus background | Cytoplasm source |
| --- | --- | --- |
| A1 | h_2_s | h_2_s |
| A2 | h_2_s | Zhenshan97A |
| A3 | h_2_s | D702A |
| A4 | h_2_s | G46A |
| A5 | h_2_s | K18A |
| A6 | h_2_s | XieqingzaoA |
