# Supplementary material for: Analysis of Cytoplasmic Effects and Fine-Mapping of a Genic Male Sterile Line in Rice
Source: PLoS One. 2013 Apr 16;8(4):e61719. doi: 10.1371/journal.pone.0061719 (PMC3628577; doi:10.1371/journal.pone.0061719)
Supplement: Table S2 — h2s cytoplasmic effects on seed set rate, weight/panicle and yield in comparison with Zhenshan97A (A2), D702A (A3), G46A (A4), K18A (A5) and XieqingzaoA (A6). (DOCX) [file pone.0061719.s010.docx]

**Table S2** *h_2_s* cytoplasmic effects on seed set rate, weight/panicle and yield in comparison with Zhenshan97A (A2), D702A (A3), G46A (A4), K18A (A5) and XieqingzaoA (A6).

| Paired comparison | Plant height  (cm) | effective panicle No. | 1,000-grain weight (g) | Grain No./panicle |
| --- | --- | --- | --- | --- |
|  |  |  |  |  |
| A1/R1 VS A2/R1 | 5.67**/7.92* | 0.45/0.43 | 0.98/-0.47 | -0.56/-6.70 |
| A1/R1 VS A3/R1 | 3.40/4.06* | 0/-0.33 | 0.15/-2.13* | -8.19/-16.23* |
| A1/R1 VS A4/R1 | 2.83/-0.67 | -0.13/-0.29 | 0.3/-0.16 | -8.11/-17.88** |
| A1/R1 VS A5/R1 | 2.50/0.16 | -0.97/-0.99 | 0.54/-1.02 | 9.25/-2.89 |
| A1/R1 VS A6/R1 | 1.60/-0.54 | -0.42/-0.17 | 0.87/-0.61 | -4.09/-4.12 |
| A1/R2 VS A2/R2 | 1.41/-0.58 | 0.12/0.93 | 0.50/-0.31 | -1.51/5.17 |
| A1/R2 VS A3/R2 | 3.98*/0.64 | 0.25/0.68 | 0.11/0.04 | 4.51/2.40 |
| A1/R2 VS A4/R2 | 2.11/-2.75 | 0.50/0.64 | 1.00/1.15 | 4.18/13.56* |
| A1/R2 VS A5/R2 | 2.91/-3.62 | 0.28/0.70 | 0.78/1.15 | -7.19/1.97 |
| A1/R2 VS A6/R2 | 1.76/0.34 | -0.21/-0.31 | 0.62/0.35 | 2.75/9.51 |
| A1/R3 VS A2/R3 | -1.59/1.14 | -0.30/-0.42 | -0.53/-0.94 | 11.93*/10.38* |
| A1/R3 VS A3/R3 | -1.66/-4.78 | -0.05/0.53 | -1.72*/-1.88* | 7.49/-0.21 |
| A1/R3 VS A4/R3 | -0.66/1.18 | 0.73/0.81 | -0.34/-0.75 | -4.83/-21.51** |
| A1/R3 VS A5/R3 | 1.14/1.83 | 0.20/-0.07 | -0.03/0.03 | 1.18/-0.55 |
| A1/R3 VS A6/R3 | 0.81/3.74 | 0.05/-0.34 | 0.14/0.68 | 0.48/-12.54* |
| A1/R4 VS A2/R4 | 0.87/-3.14 | -0.20/-0.16 | -0.16/-1.53* | -4.42/-16.98* |
| A1/R4 VS A3/R4 | 1.03/1.09 | 0.17/-0.24 | -0.27/-1.19 | 5.98/2.99 |
| A1/R4 VS A4/R4 | 0.66/-4.86 | 0.24/0.21 | -0.49/-1.40 | -12.49*/-7.26 |
| A1/R4 VS A5/R4 | 1.57/-3.57 | 0.22/0.45 | -0.49/-0.99 | 8.52/-7.09 |
| A1/R4 VS A6/R4 | 1.06/5.26* | -0.20/-0.46 | -0.77/-0.90 | 5.15/0.85 |
| A1/R5 VS A2/R5 | 3.73*/12.28** | -0.30/0.11 | 0.89/-0.77 | -11.21*/-14.40* |
| A1/R5 VS A3/R5 | 4.10*/1.89 | -0.70/0.03 | 1.28*/1.24* | 2.62/6.64 |
| A1/R5 VS A4/R5 | 3.67/3.74 | -0.77/-0.61 | 1.00/0.87 | -12.56*/-13.88* |
| A1/R5 VS A5/R5 | 3.57/3.30 | 0.02/-0.29 | 0.83/0.37 | -14.09**/-11.42* |
| A1/R5 VS A6/R5 | 2.03/11.20** | -0.42/-0.75 | 1.08/-0.28 | -6.92/-10.37* |
| Percentage of comparisons with significant effect (%) | 16/20 | 0/0 | 8/16 | 20/44 |

The numbers separated by slash the遗传与发育 of the data collected from 2006 and 2008represent the data of the year 2006 (left) and 2008 (right). * and ** Significant at 0.05 and 0.01 level, respectively.
