# Supplementary material for: Analysis of Cytoplasmic Effects and Fine-Mapping of a Genic Male Sterile Line in Rice
Source: PLoS One. 2013 Apr 16;8(4):e61719. doi: 10.1371/journal.pone.0061719 (PMC3628577; doi:10.1371/journal.pone.0061719)
Supplement: Table S3 — GCA effects on seed set rate, weight per panicle and yield among cytoplasms of h2s (A1), Zhenshan97A (A2), D702A (A3), G46A (A4), K18A (A5) and XieqingzaoA (A6). (DOCX) [file pone.0061719.s011.docx]

**Table S3** GCA effects on seed set rate, weight per panicle and yield among cytoplasms of *h_2_s* (A1), Zhenshan97A (A2), D702A (A3), G46A (A4), K18A (A5) and XieqingzaoA (A6).

| Parents | Plant height  (cm) | effective panicle No. | 1,000-grain weight (g) | Grain No./  panicle |
| --- | --- | --- | --- | --- |
| A1 | 1.62a/1.68a | -0.05a/0a | 0.21b/-0.32b | -1.07bc/-3.69c |
| A2 | -0.40b/-2.08b | 0a/-0.17a | -0.13b/0.49a | 0.08b/0.82b |
| A3 | -0.55b/-1.50b | 0.02a/-0.13a | 0.30a/0.47a | -3.55c/-2.80b |
| A4 | -0.10b/2.35a | -0.16a/-0.14a | -0.09b/-0.26b | 5.69a/5.71a |
| A5 | -0.72b/1.86a | 0a/0.04a | -0.12b/-0.22b | -0.60b/0.31b |
| A6 | 0.17b/-2.32b | 0.19a/0.41a | -0.18b/-0.16b | -0.54b/-0.35c |

The numbers separated by slash the遗传与发育 of the data collected from 2006 and 2008represent the data of the year 2006 (left) and 2008 (right). Values followed by the same letter are not significantly different. Lower-case letters indicate significant differences at *P*=0.05.
